# Biomechanical and Cortical Control of Tongue Movements During Chewing and Swallowing

**DOI:** 10.1007/s00455-023-10596-9

**Published:** 2023-06-16

**Authors:** Callum F. Ross, J. D. Laurence-Chasen, Peishu Li, Courtney Orsbon, Nicholas G. Hatsopoulos

**Affiliations:** 1 Department of Organismal Biology & Anatomy, The University of Chicago, 1027 East 57th St, Chicago, IL 60637 USA; 2 Department of Radiology, University of Vermont Medical Center, Burlington, USA; 3 National Renewable Energy Laboratory, National Renewable Energy Laboratory, Golden, Colorado, USA

**Keywords:** Mastication, Deglutition, XROMM, Cortex, Motor control

## Abstract

Tongue function is vital for chewing and swallowing and lingual dysfunction is often associated with dysphagia. Better treatment of dysphagia depends on a better understanding of hyolingual morphology, biomechanics, and neural control in humans and animal models. Recent research has revealed significant variation among animal models in morphology of the hyoid chain and suprahyoid muscles which may be associated with variation in swallowing mechanisms. The recent deployment of XROMM (X-ray Reconstruction of Moving Morphology) to quantify 3D hyolingual kinematics has revealed new details on flexion and roll of the tongue during chewing in animal models, movements similar to those used by humans. XROMM-based studies of swallowing in macaques have falsified traditional hypotheses of mechanisms of tongue base retraction during swallowing, and literature review suggests that other animal models may employ a diversity of mechanisms of tongue base retraction. There is variation among animal models in distribution of hyolingual proprioceptors but how that might be related to lingual mechanics is unknown. In macaque monkeys, tongue kinematics—shape and movement—are strongly encoded in neural activity in orofacial primary motor cortex, giving optimism for development of brain–machine interfaces for assisting recovery of lingual function after stroke. However, more research on hyolingual biomechanics and control is needed for technologies interfacing the nervous system with the hyolingual apparatus to become a reality.

## Introduction

In humans lingual dysfunction is associated with a wide range of disorders including spasmodic dysphonia [1, 2], dysphagia [3–10], orofacial dystonia and dysarthria [11], apraxia of speech [12–14], obstructive sleep apnea [15, 16] and cortical strokes [17–26] Dysphagia also affects 60–75% of head and neck cancer patients [27], as a result of either the cancer itself, or of surgical, chemotherapeutic or radiation treatments [28], impacting quality of life [29, 30]. Rehabilitation or cures for these dysfunctions depend on a solid understanding of how tongue movements are produced and controlled both biomechanically and by the central nervous system. To date our understanding of the control of mammal tongue movements has been limited by lack of high resolution 3-dimensional (3D) data [31]. Recent work in multiple labs deploying the XROMM workflow (X-ray Reconstruction of Moving Morphology) for analysis of biplanar videoradiographic data [32] has seen significant progress in measurement of tongue kinematics in pigs and primates [33–40]. The last few years have also witnessed a burgeoning number of studies relating tongue function to neuronal activity in cerebral cortex. Previous reviews have emphasized the role of oral and pharyngeal reflexes in chewing and swallowing [41], coordination of upper airway muscles during swallowing and breathing [42], and brainstem neuronal networks in control of tongue movement [43, 44]; however, accumulated evidence reviewed here suggests that orofacial sensorimotor cortex (OSMcx) also plays an important role in control of tongue movement during chewing and swallowing [20, 45–50]. Indeed, cortex may be more important in control of tongue than jaw movements during feeding. Our understanding of the biomechanical and cortical control of tongue movements is also being improved by computational modeling of the human tongue by several groups [51–59]. Our lab has pioneered simultaneous recordings using both multi-electrode arrays in OSMcx and high speed digital videoradiography, first in 2D [60, 61] and now in 3D using XROMM [35, 40]. Other labs are investigating the role of cortex in control of tongue movements in mice, although at present mouse tongue tip kinematics have only been measured outside of the mouth [62]. Together, these new methods for measuring 3D tongue movement, for recording from cerebral cortex, and for modeling tongue biomechanics are providing unprecedented insight into how jaw and tongue movements are controlled, opening up new horizons in the study of orofacial neuroscience.

Here we review the evidence on tongue kinematics during chewing and swallowing, the biomechanical mechanisms thought to produce those movements, and the role of cortex in their control. We focus on chewing and swallowing of solid food because those tongue kinematics are larger, more complex and more asymmetrical than those during drinking and speech [31, 39, 63]. We emphasize studies of adult animals; excellent studies of hyolingual kinematics in infant animals are beyond the scope of this review [64–75]. Emphasis is placed on the role of cortex in control of tongue movements because our recent work in macaques reveals close correlations between cortical activity and tongue shape during feeding [40]. Moreover, the superficial location of sensorimotor cortex makes it relatively accessible for minimally or non-invasive treatment modalities, such as transcranial magnetic stimulation, or implantable brain-machine interfaces [76]. We emphasize results from macaque primates because similarities to humans make them an ideal nonhuman primate (NHP) model of biomechanics and motor control of human feeding system function and dysfunction [77–90]. Macaques resemble humans in connectivity between brain regions involved in orofacial behaviors [91–94], and, as reviewed below, they also closely resemble humans in jaw & hyolingual anatomy, kinematics, & muscle activity during chewing and swallowing [33, 34, 60, 81, 82, 95–97]. However, we also review and discuss important studies of hyolingual biomechanics and control of tongue function in other animal models, including rats, rabbits, pigs and cats [38–39, 62, 98–101]. Similarities and differences between humans and animal models can inform selection of animal models for research into specific aspects of hyolingual control.

We first review the anatomy of the jaws and hyolingual apparatus in humans, macaques and other animal models, then we summarize available data on hyolingual kinematics during feeding (stage 1 transport, chewing, stage 2 transport and swallowing) before reviewing hypotheses about the biomechanical mechanisms driving tongue protraction and retraction, twisting and flexion during chewing, and tongue base retraction during swallowing. We then review evidence for sensory modulation of tongue movements and the role of cortex in swallowing and tongue movements.

### Morphology of Jaws and Hyolingual Apparatus

Experimental studies using animal models provide invaluable insights into healthy and pathophysiological hyolingual function in humans [37]. With a wide array of model species available, choosing the right model for a given research question requires some basic understanding of the anatomy and behavior of the model organism. Macaque and other anthropoid primates are most similar to humans in feeding system morphology and function, but non-primate animal models are useful when morphological similarities to humans are not necessary to address the question at hand. Here we summarize our current knowledge on the hyolingual musculoskeletal anatomy in macaques and other commonly used animal models to better inform physicians, researchers, and other clinicians on choosing the appropriate models for experimental investigation.

#### Jaws

The anatomy of the macaque feeding system closely resembles that of humans in several important respects [102]. As in adult humans, the mandibular symphysis of adult macaques is solidly fused into a single bone and their chewing involves significant mediolateral components of jaw movement during the power stroke of mastication. This is also the case in rabbits and pigs. In contrast, the symphyses of rats, cats, and dogs, are not fused in adults, allowing various degrees of independent movements of hemimandibles that are not human-like [103–105]. Rats use anteroposterior jaw movements during chewing, and cats and dogs use scissor-like, primarily vertical jaw movements [106, 107]. The extent to which variation in jaw movement profile impacts tongue kinematics and coordination remains to be established. The hard palate of macaques resembles that of humans in lacking prominent transverse rugae, which impacts tongue function during intra-oral food transport, and the squeeze-back mechanism of transport to the oropharynx [81].

#### Tongue

**Fig. 1:**
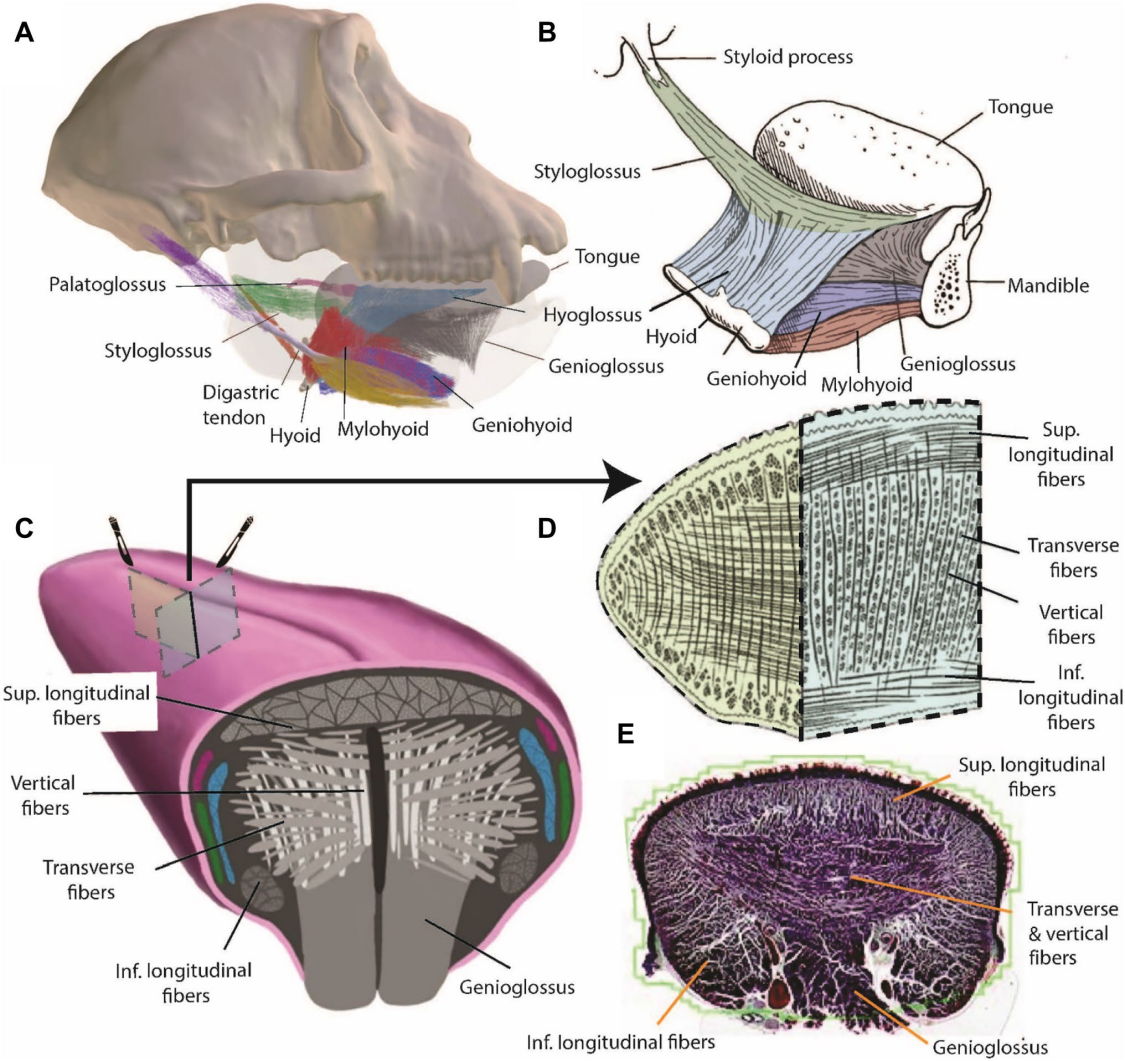
Hyolingual muscles in macaque closely resemble those of humans. **A, B** Show right lateral views of suprahyoid and extrinsic lingual muscles in macaque (**A**) and humans (**B**). Digastric and palatoglossus not shown in (**B**). **C** Shows posterior oblique view of a macaque tongue, with a coronal section showing intrinsic lingual muscles and extrinsic lingual muscles through the genioglossal “stem”. Extrinsic lingual muscle color scheme follows (**A, B**). **D** Coronal + sagittal wedge from anterior tongue blade of macaque. **E** Coronal section from human tongue blade showing intrinsic muscle layout. **A, C** Modified from [33, 34]; **B, E** modified from [42], **D** Modified from [113]

Humans and macaques also share a common Bauplan of tongue anatomy (Fig. 1) [33, 34, 108]: a spatulate, fleshy tongue composed of a core of intrinsic tongue muscles, interweaving vertical and transverse muscle fascicles, capped by a layer of superior longitudinal muscles [34, 108–110]. The genioglossus forms an inferior stem that interweaves with the intrinsic muscles; on either side of that stem are the paired inferior longitudinal muscles. Other extrinsic lingual muscles, stylo-, hyo-, and palatoglossus, connect the tongue to cranium and hyoid, merging with intrinsic muscles on the sides of the tongue. Human tongue muscle volumes have been quantified [111], but those of macaques and other animal models have not, so claims that humans have more intrinsic tongue muscles than other primates remain to be confirmed [112].

The macaque tongue closely resembles that of humans in being dorsoventrally deeper—from mylohyoid to the palatal surface—than that of many other mammals, possibly related to the anterior position of the hyoid (Fig. 1). Consequently, both macaques and humans have small valleculae that limit the amount of food that can accumulate there before a swallow [82]. The tongue forms the floor of the oral cavity and extends posteriorly through the fauces to also form the anterior wall and floor of the oropharynx [114]. The surface of the posterior one-third of the tongue—the tongue base—forms a wall facing posteriorly into the oropharynx in both macaques and humans, although this wall is more vertical and relatively taller in humans because the hyoid is positioned lower and the oropharynx is deeper [102]. Macaque tongue muscles resemble humans in having a higher proportion of slow fiber types than in rats and cats [115]. Macaque jaw and extrinsic tongue muscles are similar to those of humans, including a truly two-bellied digastric muscle, although the macaque digastric is only indirectly connected to the hyoid via a broad fascial connection, not a discrete sling for the digastric tendon as in humans. Styloglossus in macaques arises from the stylomandibular ligament rather than the styloid process and hence is more horizontally oriented than in humans [116] (Fig. 1A, B), but its relative proportions of fast, slow and hybrid fiber types are similar in the two species [117].

The mammalian tongue is surrounded by a variably keratinized, stratified squamous epithelium resting on a dense connective tissue sheath, the material properties of which are important for tongue function but poorly studied [110, 118, 119]. The epithelial surface of the tongue in macaques and humans is marked by dense filiform papillae on the tongue’s dorsal surface, with interspersed fungiform papillae and a V-shaped array of vallate papillae anterior to the sulcus terminalis, which separates the anterior two-thirds from the posterior third of the tongue [112]. Like that of humans, the muscular tongue base of macaques is overlain by dense glandular and tonsillar tissue.

#### Hyoid

**Fig. 2:**
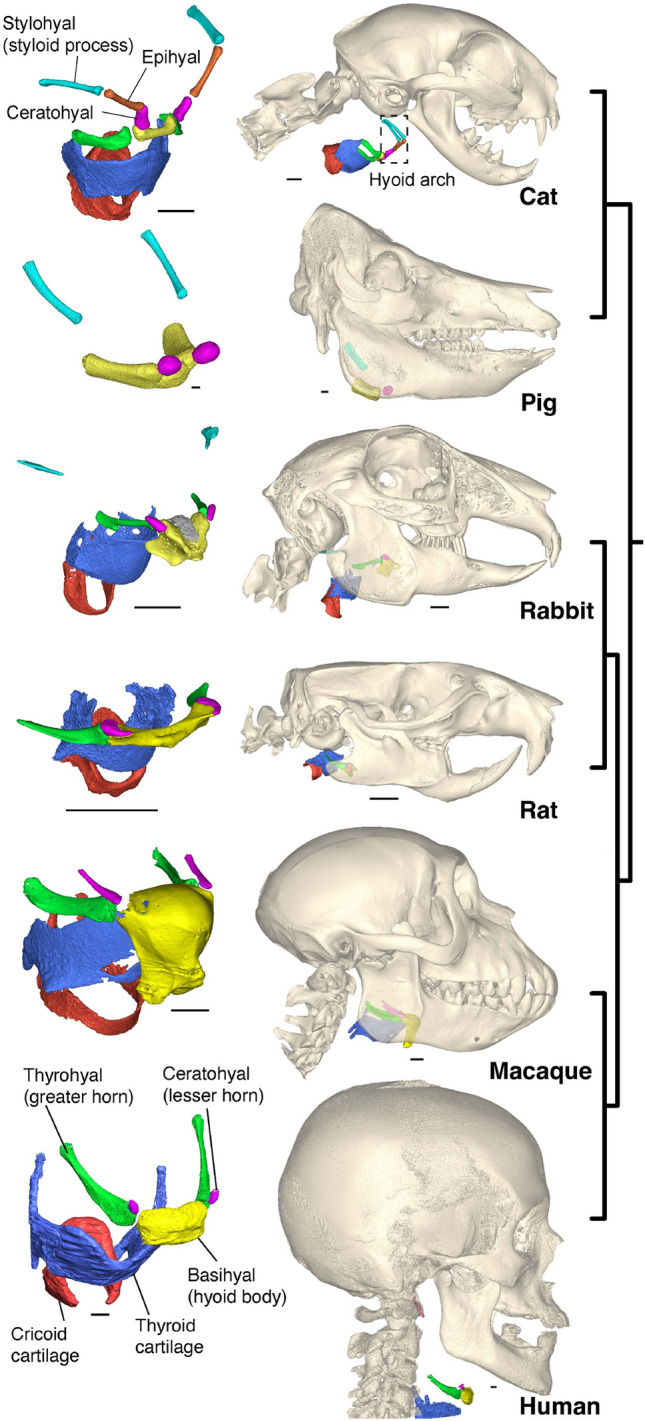
Hyoid chain morphology in humans and animal models of chewing and swallowing. Right column, location of hyoid and larynx in lateral view. Left column, left oblique anterior view of hyoid chains. Cats and dogs have integro-cornuate hyoid chains; the other species are discrete-cornuate in various ways. The human hyoid is located below the bottom of the mandible at rest, like that of cats. Pig and rat 3D models are based on fresh frozen cadaveric specimens; other 3D models are based on fixed specimens. Scale bar is 5 mm

In mammals the bones of the hyoid arch partially frame the narrow, muscularized pharynx near the boundary between oropharynx and hypopharynx [120]. The degree to which hyoid chain elements are present, the shape of the basihyal, and the position of the hyoid relative to the oral cavity and mandible vary across species [114, 121–123], including among animal models commonly used to study swallow function [37, 124] (Fig. 2). The posterior cornu (greater horn in human anatomy literature) consists of the thyrohyal. The hyoids of humans and macaques, like those of pigs [125], rabbits [98], and rats [126], are ‘‘floating’’, i.e., the hyoid chain (or anterior cornu) is incomplete (discreto-cornuate sensu [123], excepting known anatomical variants in humans, e.g., Eagle syndrome), and they are connected to the basicranium only by ligaments and muscles. The diminutive ceratohyal in human is commonly termed the lesser horn. In contrast, the hyoids of other mammals, such as dogs and cats, are integro-cornuate—the basihyal is connected to the basicranium by a complete series of hyoid arch bones or cartilages (monotremes, lemurs, cats, and dogs) [121, 123, 127]. The functional significance of this variation in degree of hyoid chain completeness is not clear. In humans, abnormal ossification of hyoid arch (i.e. Eagle syndrome) can be associated with dysphagia [128]. It has been suggested that the “floating hyoid in monkeys and rabbits facilitates control of larynx position during extreme neck movement during locomotion” [98, 114]. However, humans, monkeys, and rabbits do not share greater head movements during locomotion than other mammals [129, 130], so this is unlikely to be the reason for loss of hyoid chain ossifications.

The morphology of the basihyal is notable in macaques because it is thought to function in a piston-like manner as part of a hydraulic mechanism driving tongue base retraction during swallows [33]. Monkeys, like apes and rabbits, have dorsoventrally expanded basihyals, contrasting with the more bar-like basihyals of humans, cats, dogs, and rats [98, 121, 122]. The basihyal is deeper in anthropoid primates with laryngeal air sacs (apes, macaques and baboons) than in species—like humans—that lack air sacs [122, 131]. The morphology of the thyrohyals is also of interest because of their close relationship to the laryngeal inlet and piriform sinuses [120, 121, 123]. Comparative studies employing high resolution methods for both morphological analyses, such as diffusible iodine-based contrast-enhanced computed tomography (DiceCT), and 3D kinematic analyses (XROMM) are needed to determine whether different hyoid morphotypes in different animal models are associated with differences in swallowing mechanics [34].

The position and orientation of the hyoid at rest vary between adult mammals (Fig. 2). In humans the body of the hyoid bone (the basihyal) lies 20–30 mm below the base of the mandible, as it does in cats; in pigs and rats the basihyal lies at the level of the base of the mandible; in rabbits, macaques, and capuchin monkeys the basihyal lies superior to the base of the mandible. The functional significance of this variation is currently unknown (Fig. 2) [132], but an accumulating body of evidence suggests resting hyoid posture co-varies with hyoid morphology [121], craniomandibular morphology [133], and tongue dimensions. However, teasing apart the covariation between these traits is complicated by the fact that hyoid posture also varies within individuals as a function of head flexion, as documented in humans [134], dogs [133], horses [135], opossums [136] and probably other animal models. The influence of head posture on hyoid position forms the anatomical basis for the chin-tuck maneuver in humans [137–140], but the efficacy of this maneuver varies across patient groups with different dysphagia severity and etiology [141]. A better understanding of the functional impact of head posture on swallowing performance in animal models may provide insights into improving the chin-tuck maneuver in humans. Existing evidence suggests head flexion-induced variation in hyoid position changes the dimensions of the oropharynx [140] and anteroposterior length of the laryngeal inlet [139], and may affect the force-generation capacity, shortening distance, and activity of suprahyoid muscles by altering their positions along the length-tension curve [132, 142]. Consequently, head posture should be carefully controlled and quantified in swallowing studies using animal models and humans alike.

**Fig. 3:**
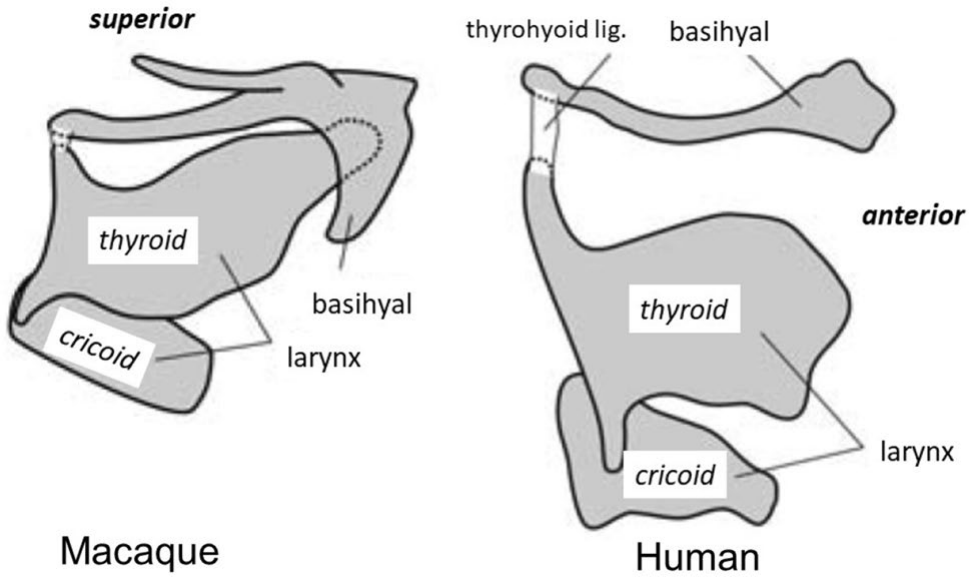
Right lateral view showing relative positions of hyoid and laryngeal cartilages (*thyroid* and *cricoid*) in humans and macaques. Modified from [143]

The position of the larynx relative to the hyoid also varies among mammals. Macaques differ from humans in having a thyroid cartilage positioned close to and overlapping with the hyoid, and the superior margins of the hyoid and larynx are positioned above the base of the mandible (Figs. 2, 3) [116, 143]. In contrast, in adult humans the hyoid lies 20–30 mm below the mandible [144], and the thyroid notch is two finger breadths below the hyoid [145]. In humans and apes, the larynx descends relative to the hyoid during infancy, then during the juvenile period both larynx and hyoid descend relative to the palate [143]; in humans the hyoid and larynx descend even further. The resulting low position of the larynx relative to the hyoid in humans is not an adaptation for speech because the larynx is also descended, either facultatively or permanently, relative to the hyoid in great apes [143] fallow deer [146], big cats [147], koalas [148], and certain bats [149], none of which speak. Moreover, despite their high larynx and hyoids, macaque and baboon vocal tracts are capable of producing all the vowel sounds needed for human speech [150]. The origin of speech during human evolution is instead associated with changes in innervation of the laryngeal muscles, the size of the hypoglossal nerve, and changes in cortical control of the tongue [151–154]. The low position of the thyroid cartilage relative to the hyoid in humans might make it possible for laryngeal elevation relative to the hyoid to contribute to epiglottal folding over the laryngeal inlet during swallowing [143, 144, 155], but macaques safely swallow solids and liquids with epiglottal inversion and minimal movement of thyroid relative to the hyoid [60, 156, 157]. The mechanics of epiglottis inversion in humans may be different from those in other mammals, or the relative position of thyroid cartilage and hyoid may not be functionally linked to epiglottis inversion at all. Comparative studies of the mechanisms of epiglottal inversion in animal models of swallowing are clearly needed.

#### Suprahyoid Muscles

The hyoid bone of mammals is connected to the mandible by the anterior digastric, geniohyoid, and mylohyoid muscles; the hammock-like mylohyoid muscle also floors what we have termed the ‘oral volume’, the oral cavity sensu stricto plus tissues between oral mucosa and mylohyoid [33]. Suprahyoid muscle morphology varies across animal models of swallowing. Rabbits, cats, dogs, and pigs only have a single belly of the digastric with no or only minimal attachment to the hyoid. Rabbits have only an anterior belly [98], cats and dogs have a single belly consisting of fused anterior and posterior bellies, but it attaches to the mandible directly, without any direct connection to the hyoid [158, 159]. Macaques and rats resemble humans in having a digastric sensu stricto attached to the hyoid by connective tissue, albeit in different ways (Table 1). Differences in digastric attachment to the hyoid probably impact the way that digastric function impacts hyoid kinematics during feeding (see below) suggesting that better studies of digastric function in animal models would be of interest.

**Table 1:** Animal models of hyolingual function compared with humans

Taxon	Basihyal craniocaudal position relative to mandible base*	Basihyal mesiodistal position relative to toothrow [121]	Basihyal shape [121, 122]	Hyoid arch completeness [121, 123]	Digastric morphology	Tongue twisting during chewing	Midline tongue base retraction during swallow
Human	20–30 mm below [144]	Near back of toothrow	Bar	Incomplete	Digastric; connected to hyoid by loop [160]	Yes [161–163]	Yes
Macaque	Above	Near back of toothrow	Shield	Incomplete	Digastric; tendinous arcade attaches to hyoid [164]	Yes [36, 82]	Yes [33]
Rat	At or above	Far behind	Bar	Incomplete	Digastric; tendinous arcade attaches to hyoid [165–167]	Unknown	Yes [168]
Rabbit	Above	Near back of toothrow	Shield [98]	Incomplete	Anterior belly only; no hyoid attachment [98]	Inferred [169]	Unknown
Cat and dog	Below	Very far behind	Bar	Complete	Fused anterior and posterior bellies; no hyoid attachment [158, 167, 170–172]	Inferred for *Felis* [100]	Yes [173, 174]
Pig	At or above	Far behind	Bar	Incomplete	Posterior belly; no/minimal hyoid attachment [175, 176]	Yes [39]	Yes [156, 177]
Opossum	Above	Far behind	Bar	Incomplete	Digastric; tendinous arcade attaches to hyoid [167, 178]	Unknown	Yes (pers. observations)

*See Fig. 2

### Hyolingual Kinematics

**Fig. 4:**
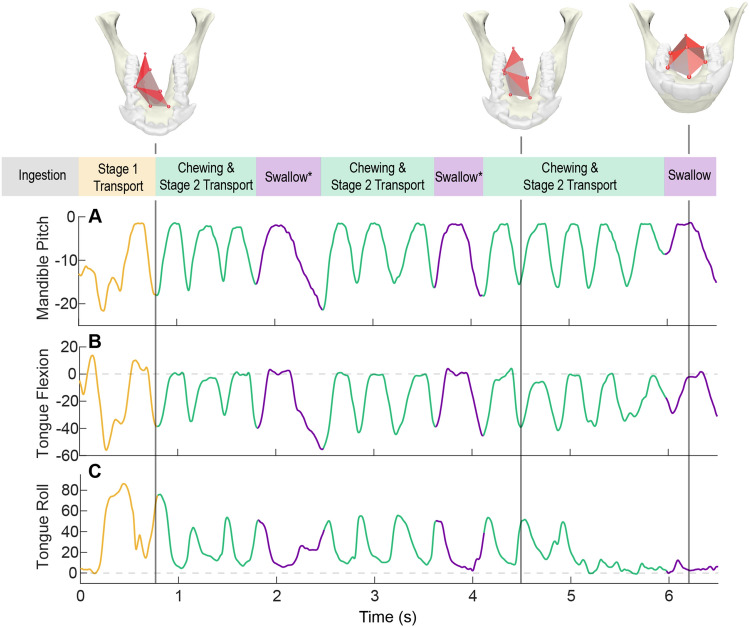
Tongue and jaw movements during a representative grape feeding sequence. Mammal feeding sequences consist of a series of gape, tongue and hyoid movement cycles that can be arranged into stages. In primates, including humans, stage 2 transport occurs during chewing cycles. **A** Mandible pitch, **B** Tongue sagittal flexion, **C** Tongue roll. Color of lines indicates the process being performed: yellow, manipulation/stage 1 transport; green, rhythmic chewing; purple, swallowing. Images above plots are stills from XROMM animations at the time point indicated by the vertical grey lines. Note that the tongue posture at maximum gape during Stage I transport and rhythmic chewing is similar. Intercalated swallows marked with *. Modified from [35]

The most important functions of the tongue in feeding are *transport* of food through the oral cavity [179], taste sensation, and *stereognosis*, or collection of somatosensory information about the location and external properties of the food [180, 181]. Stereognosis is vital for determining whether the food bolus is ready to be transported to the molars for chewing (stage 1 transport), or to the oropharynx prior to or during a swallow (stage 2 transport) [182]. Stereognosis is also important for feed-forward control of swallow kinematics [183]. During feeding the tongue moves cyclically in coordination with mandible and hyoid (Fig. 4) [99, 179, 184–186]. The internal location of tongue and hyoid makes it difficult to measure their movements during feeding with the large sample sizes needed to address drivers of variance in tongue kinematics [187]. These difficulties can be ameliorated by use of video–radiography, but until recently most such studies were single plane (2D), and mostly in lateral view; attempts to capture mediolateral (ML) tongue movements relied on non-simultaneous anteroposterior (AP), posteroanterior (PA), or dorsoventral (DV) views via radiographic or light cameras [60, 81, 82, 84, 161, 162, 184, 188–191]. However, the cumulative radio-opacity of teeth and skull in AP/PA view, the impracticality of DV views in humans, and the small gapes at which chewing occurs mean that descriptions of ML tongue movements during chewing in humans and other mammals have been coarse and qualitative [163]. Deployment of biplanar videoradiography and the XROMM workflow to study animal models has enabled collection of high resolution and high speed 3D measurements of jaw and tongue kinematics using non-standard views [33–39, 192], and development of methods for increasing the rate of marker tracking [193] and automation of kinematic analysis have significantly increased sample sizes of tongue movements [35, 38–40]. Here we present an integrated review of published 2D and 3D studies, focusing on feeding in humans, macaques, and pigs, but with reference to studies in other mammals when appropriate. We focus on intra-oral tongue function: we exclude ingestion sensu stricto—bringing food into the oral cavity or vestibule through the oral fissure—because humans and other anthropoid primates use their hands rather than their tongues to place food in the oral cavity [112].

Patterns of coordination—relative timing—of mandible, tongue, and hyoid movements vary during the feeding sequence. These patterns are quantified by measuring hyoid and tongue kinematics relative to the cyclic elevation and depression of the mandible, the gape cycle. The gape cycle is traditionally divided into four phases defined by maxima and minima in the jaw’s vertical position, velocity, and acceleration [39, 184, 194, 195]. Fast close (FC) begins at maximum gape and ends as the teeth come into contact with the food; slow-close (SC), the phase when most food break-down occurs, begins as tooth-food-tooth contact slows jaw elevation velocity. SC ends and slow-open (SO) begins at minimum gape; SO ends at the start of fast open (FO), as jaw depression velocity rapidly increases. Fast-open (FO) ends at maximum gape, at which point a new cycle begins with FC. The phase boundaries also correspond to significant changes in sensorimotor events, as reflected in changes in firing rates of sensory neurons [196] and changes in tongue movement trajectories. The phases of the gape cycle vary depending on the mechanical properties of the food bolus, on feeding behavior—licking, lapping, drinking, eating—and on the dominant function in any cycle, such as bolus manipulation, chew, or swallow.

The tongue translates along all three axes—protraction-retraction, elevation–depression, right-left displacements—in combination with complex 3D shape changes (Fig. 6). Shape is the geometric information remaining when translational, rotational, and scaling effects are removed [197]. Geometric morphometric techniques for studying shape have been used to study hyoid kinematics in humans [198] and tongue shape in macaques [40]. However, distinguishing between tongue shape change and displacement has limited utility, as deformation in a biomechanical context (i.e., in a cranial or mandibular coordinate system) will invariably displace some parts of the tongue relative to the skull; a given tongue movement is typically achieved through a combination of 3D shape change and regional- or whole-tongue displacement. In virtually all cases, the displacement combines deformation and pure translation. The notion that extrinsic muscles displace the tongue and intrinsic muscles change its shape [199] has long been rejected [200–202]. Here we review jaw, tongue, and hyoid movements during stage 1 transport (defined below), chewing, and swallowing of solid foods. We then discuss potential biomechanical mechanisms of hyoid and tongue movement, drawing on data from drinking when needed, before reviewing evidence for the role of sensory feedback and cortical activity in control of jaw and tongue movement.

#### Stage 1 Transport

**Fig. 5:**
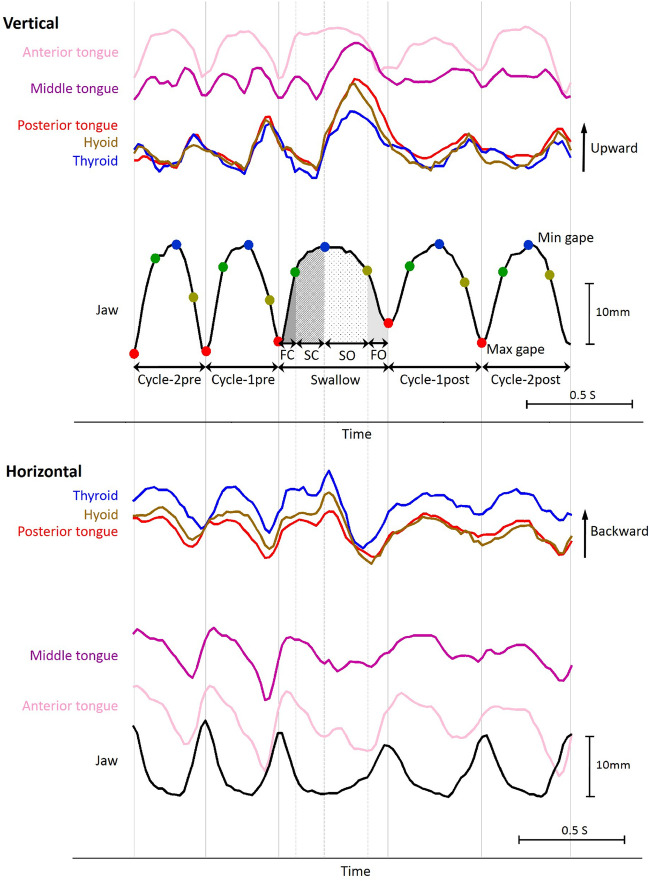
Vertical and horizontal (2D) tongue and hyoid movements in a macaque during swallows and adjacent cycles, from [60]. The four phases of the gape cycle are shown: fast close (FC), slow close (SC), slow open (SO) and fast open (FO)

**Fig. 6:**
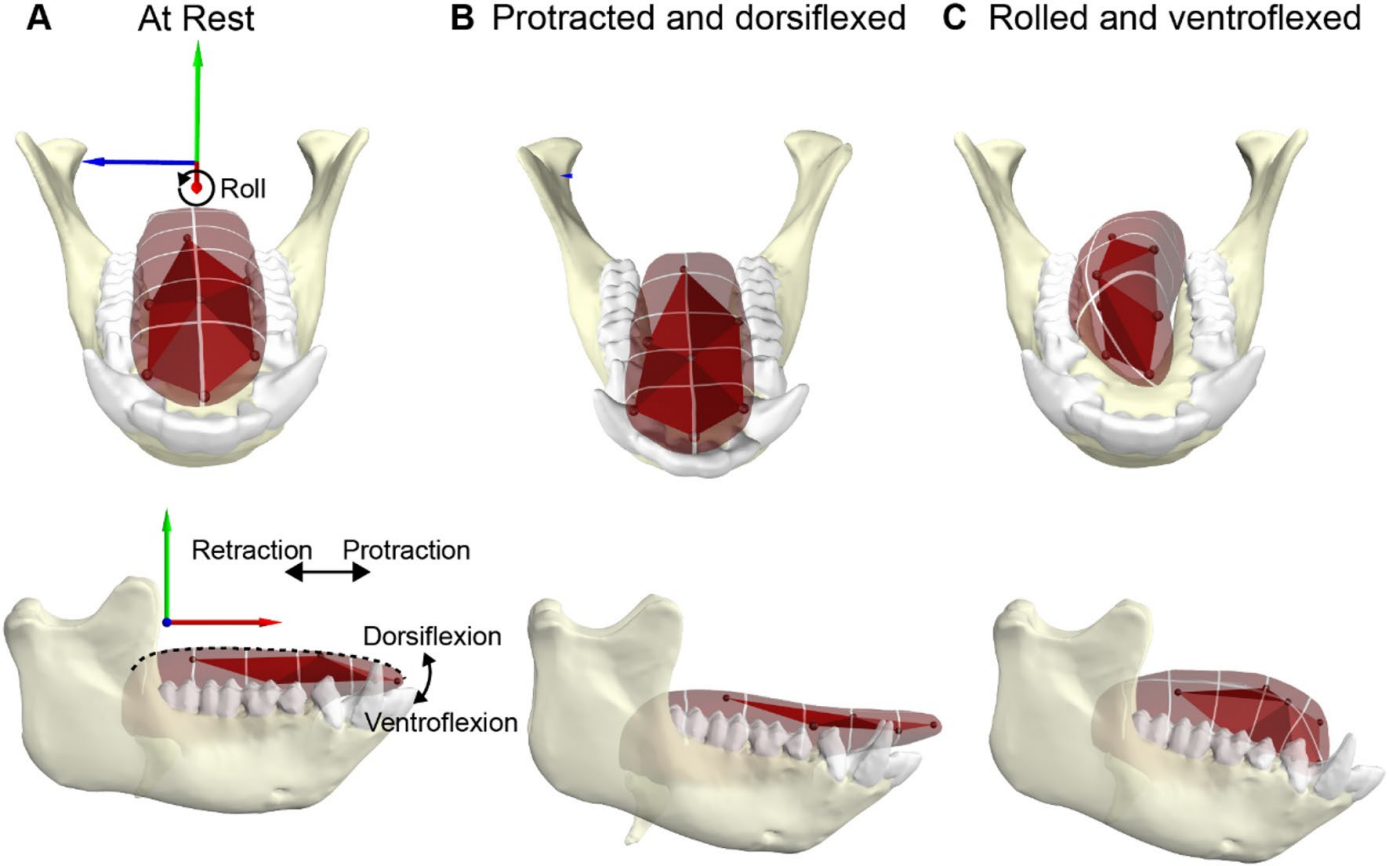
Definition of cranial coordinate system and tongue motions. For each posture, mandibular and tongue marker positions correspond to in vivo positions extracted from different time points of a single feeding sequence using XROMM. The semi-transparent tongue body mesh was sculpted in Autodesk Maya based on the known depth of the markers. Basihyoid position based on the position of a hyoid marker. **A** Tongue at rest. The cranial coordinate system (red, green, and blue axes) in which the tongue moves has its origin at the posterior nasal spine (not shown), and the A-P axis is oriented in line with the maxillary tooth row (not shown). Protraction and retraction are anterior and posterior translation (displacement) of the tongue along the A-P axis. *Protrusion* is protraction of the tongue tip past the incisors (as in panel (**B**)) [204]. Protraction and retraction are usually produced by a combination of whole-tongue translation and deformation—lengthening and shortening—within the tongue. Sagittal flexion is bending of the tongue in a plane perpendicular to the tongue surface; resulting in the raising and lowering of the tongue tip. Positive sagittal flexion is synonymous with dorsiflexion, and negative sagittal flexion is synonymous with ventroflexion. Notably, in the case of a rolled tongue, flexion may not be in the sagittal plane. **B** The tongue in protracted and dorsiflexed posture during initial ingestion. **C** The tongue shown rolled and ventroflexed, as when positioning food onto the tooth row during chewing. Note hyoid protraction associated with tongue flexion and roll in (**C**), and retraction associated with tongue protrusion in (**B**)

Stage 1 transport is the movement of food from the ingestion point to the molars. Tongue kinematics during stage 1 transport in humans are not well-documented, despite their importance for stereognosis of the food bolus [189]. In humans, macaques, pigs, and rabbits the tongue protracts during the SC and SO phases of early stage 1 transport cycles and the dorsum of the anterior tongue forms a trough to cradle the food item for transport (Fig. 6B) [81, 161]. Cats, dogs, and pigs have prominent transverse palatal rugae against which the bolus can be pressed to hold it in place as the tongue surface slides forward underneath it during SC and SO. However, humans and macaques lack prominent transverse palatal rugae, so tongue twisting is an important mechanism for pushing the bolus against the lingual surfaces of the upper teeth as the tongue slides forward during SC and SO [81]. Then, as the mandible quickly depresses then elevates during FO and FC, the bolus is moved posteriorly by simultaneous contraction of the middle and posterior regions of the tongue, as well as by rapid posterior movement of hyoid and tongue as a whole [81, 82]. The anterior, middle, and posterior tongue all move forward in phase during SC and SO, albeit with some differences in relative velocity due to differential contraction of middle and posterior tongue [203]. In macaques, stage 1 transport can involve multiple gape and tongue movement cycles, during which the food is moved forward and back, presumably for oral stereognosis (Fig. 5) [81, 82].

During later stage 1 transport cycles humans and macaques twist their tongues towards the working side, positioning the bolus between occlusal surfaces of the approaching toothrows (Fig. 6C) [81, 82, 161]. Twisting during stage 1 transport by humans was documented by Abd-el-Malek using photography of people chewing on colored candies, and by Tomura et al. using asynchronous lateral and AP cineradiography of lead markers glued to the tongue surface [162]. During stage 1 transport, twisting of the tongue to the working side reaches 23°–28° at maximum gape, with larger twisting angles in the premolar than the molar region of the tongue. In macaques, tongue twisting in stage 1 transport is also more exaggerated than in chewing, possibly because the bolus is larger (Fig. 5).

#### Mastication

Mastication (mammalian chewing) is cyclic breakdown of the food bolus between the molars. Effective chewing depends on tight coordination of lips, cheeks, jaw, and tongue modulated via sensorimotor integration of tongue movement and sensation. This is well illustrated by studies showing how in macaques tongue movement is important for triggering the start of the FO phase across three types of chewing cycle [84]. In all three cycle types tongue protraction starts around the start of SC and tongue retraction starts around the start of FO. This is true of simple chews, which lack a SO phase, so that FO and tongue retraction start at minimum gape; of simple transport cycles, which have a short SO phase followed by a pause during which the tongue moves and expands forwards before retracting during FO; and of complex chews, with a long SO phase during which the tongue continues to protract until the start of FO. In humans, anterior tongue movements are used to accumulate food bolus on the anterior palate during the SC phase of chewing cycles and later to transport the bolus posteriorly during FO [189]. Sensory information is therefore essential for triggering kinematic changes defining transitions in gape cycle phases; interruption of sensory feedback significantly impedes feeding performance.

Three-dimensional tongue movements during chewing are important for positioning the food bolus, especially in mammals that chew unilaterally, such as humans, macaques, pigs, cats, rabbits, and opossums. Abd-el-Malek showed that during chewing cycles (his ‘guarding’ stage) the middle of the tongue twists to the biting side to position or retain the food bolus between the approaching teeth [161]. Tongue twisting during chewing in humans can be inferred from lateral view radiographic data [189, 205], but is most clearly documented by PA videoradiography. Mioche et al. found that “[a]s the jaws approached maximum gape, the tongue surface rotated towards the sample, pushing it on to the dental arch during closing. The positioning was complete before tooth-food-tooth contact” (p. 274) [163]. Tomura et al. reported that tongue twisting peaked at 10°–20° during FC of mastication cycles and hypothesized that “in order to prevent particles from falling out of the occlusal surfaces of the lower molars, the tongue maintained great tortuosity and while its dorsal part on the working side was pressed on the lingual side and the alveolare [sic] region of the lower molars, that on the balancing side was upheaved to make a wall so as to prevent the food particles from escaping towards the balancing side” [162] (p. 49).

**Fig. 7:**
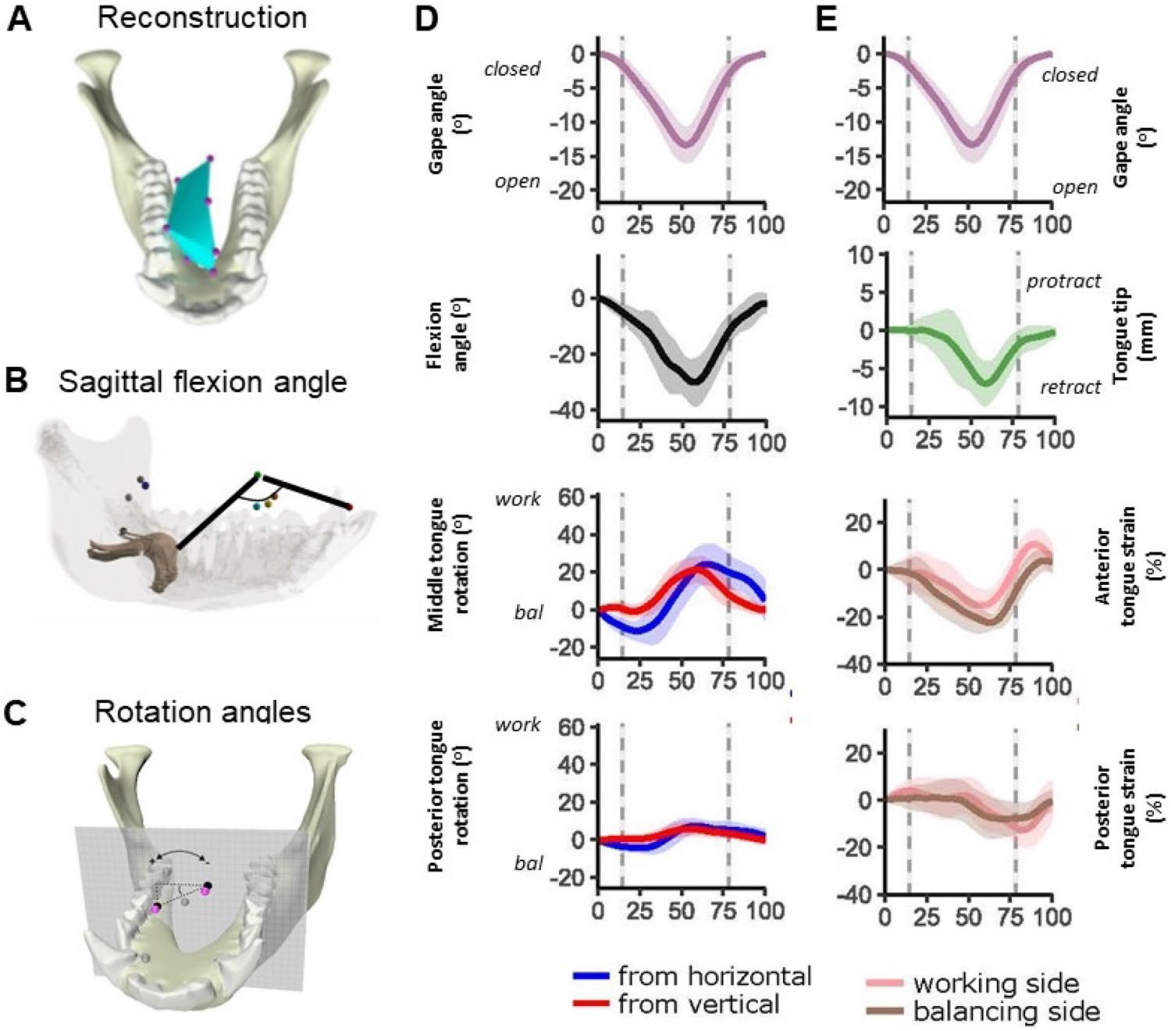
Tongue kinematics in macaques during chewing. Data derived from XROMM-based measures of 3D marker positions during grape chewing (n = 25 cycles). Data from three other individuals are very similar. **A** 3D reconstruction of tongue posture at peak flexion and roll. Markers are connected via triangular planes to approximate the tongue’s dorsum. **B** Markers used to measure sagittal flexion angle: tongue tip, superficial midline marker in middle tongue plane, and hyoid. Note that as these markers move in 3D space the plane they define rotates out of sagittal. **C** Rotation angles for middle and posterior tongue planes are measured as coronal plane projections of vertical (not shown) and horizontal lines connecting markers. **D** Plots of mean flexion and rotation angles through gape cycles normalized from 0 to 100%, with start and end of cycles being minimum gape. **E** Tongue tip protraction and retraction; strain (%) in anterior tongue, distances from lateral tongue markers in middle tongue plane, posterior tongue, distances between lateral markers in middle and posterior planes. Modified from [36]

We quantified tongue roll in macaques using rotations of marker sets placed in coronal planes through middle and posterior tongue (Fig. 7A–C) [36]. Specifically, we measured orientations of vertical lines, connecting superficial and deep midline tongue markers, and horizontal lines connecting lateral markers, projected onto coronal planes. We also quantified sagittal flexion using hyoid, middle, and anterior tongue markers, and lateral flexion using strain in distances between lateral markers in middle and posterior planes, and between lateral markers in the middle plane and a tongue tip marker. During the fast phases of the gape cycle (FO and FC) the tongue flexes in sagittal planes and the dorsum of the middle tongue twists towards the chewing side (Fig. 7D), with flexion and roll both peaking at ca. 20°–30° during FC. Balancing side flexion of the tongue tip is apparent starting in FO with the tip of the tongue usually reaching maximum lateral flexion after peak sagittal flexion (Fig. 7). In humans and macaques, rotations of the posterior tongue plane are similar in sign but smaller in magnitude (< 23°–24°) than those of the middle plane, so that working side roll of the tongue dorsum is actually due to internal twisting or torsion of the tongue, implying that there is significant shear between coronal planes through the tongue.

Review of published 2D radiographic studies suggests that the tongues of rabbits, cats, and pigs also flex and twist during chewing. In rabbits, anterior middle and posterior tongue markers all start protracting around the start of SO, but during FO the middle and anterior markers start retracting while the posterior tongue marker is still protracting [169]. As a result, tongue length shortens throughout opening, which the authors attributed to tongue sagittal flexion. In DV view, the molar region of the tongue gets narrower during SC than during FC, which the authors suggested may be due to twisting of the tongue to position the food bolus between the teeth [169]. The tongue of cats clearly flexes during FO, FC, and SC, combined with roll about some combination of AP and vertical axes [100]. In pigs, the tongue has long been argued to flex during ingestion and roll towards the chewing side during mastication [206]. Reported 3D measures of tongue deformation in pigs are compatible with flexion and twisting of the tongue during chewing, and such deformation is clearly visible peaking around the start of FC in the Supplementary Video of chewing accompanying [39]. Thus, the available data suggest that flexion and rolling of the tongue are important for bolus positioning during chewing in several species of mammals that chew unilaterally. How these shape changes are produced mechanically, and how they might be coordinated and modulated neurologically are discussed below. Three-dimensional lingual kinematics in animals, such as rats, that chew bilaterally are yet to be quantified.

#### Stage 2 Transport

Stage 2 transport is the movement of food from the oral cavity into the oropharynx either prior to or during a swallow. Most mammals drive this posterior transport via a “pull-back” mechanism, in which posterior movement of the bolus into the oropharynx is associated with retraction of hyoid and tongue during FO. Although humans can also use a “pull-back” mechanism for stage 2 transport, they most commonly use a “squeeze back” mechanism in which tongue protraction under the bolus causes the contact between the tongue surface and the palate to gradually move posteriorly, squeezing the bolus back onto the oropharyngeal surface of the tongue [189, 207]. In humans, stage 2 transport is associated with anterior and superior movement of the hyoid during SO and FO [208]. Macaques also use the “squeeze back” mechanism of stage 2 transport; i.e., posterior bolus movement into the oropharynx occurs simultaneous with hyoid and tongue protraction during SO [82]. Exactly how the food bolus is selected for stage 2 transport is unknown, but it certainly relies on sensory information from tongue and palate.

In humans and macaques stage 2 transport cycles are recognizable on the basis of a long SO phase [60], but stage 2 transport can also occur in cycles with no noticeable change in jaw movement profile [82]. Having relatively small valleculae, humans and macaques accumulate food on the pharyngeal surface of the tongue over multiple cycles, the bolus passing forward and back through the fauces as the tongue protracts and retracts during chewing. The oft-cited palatopharyngeal “seal”, separating the oral cavity from the oropharynx is thought to be important for control of liquid boluses during drinking, but it does not hold for solid food feeding in humans and macaques [189, 208].

#### Swallowing

In humans, macaques, and other mammals deglutition—transport of the food bolus from the oral cavity and oropharynx into the esophagus—occurs in three stages: an oral stage, when the food bolus is chewed, mixed with saliva, and, when suitable for swallowing, moved via stage 2 transport into the oropharynx; a pharyngeal stage, when the food bolus in the oropharynx is driven across the oropharynx by tongue base retraction and hyoid elevation; and an esophageal stage when the food is squeezed down into the stomach by peristalsis [209]. Jaw and tongue kinematics around and during swallowing are similar in humans and macaques, reflecting a shared mechanisms of stage 2 transport [60]; this mechanism allows humans and macaques to smoothly intercalate multiple swallows into a single feeding sequence, perhaps compensating for the fact that the small size of their valleculae prevents accumulation of a large bolus in the oropharynx prior to swallowing [82, 95].

Tongue and hyoid movements are essential for successful performance of the oral and pharyngeal stages of swallowing. In macaques and humans, the same squeeze-back mechanism used in stage 2 transport is also used in oral and pharyngeal stages of swallowing, albeit over longer duration and with greater displacement of the hyoid [33, 60, 82, 208]. Starting around minimum gape and continuing through SO the hyoid elevates and protracts and the contact point between the tongue and the palate slides posteriorly, squeezing the food bolus into the oropharynx where it merges with food bolus accumulated in the valleculae during prior stage 2 transport cycles. During the pharyngeal stage the hyoid continues to protract, pulling the larynx up and forward, opening up the hypopharynx and the now-relaxed upper esophageal sphincter. The human larynx also elevates relative to the hyoid, contributing to downward flip of the epiglottis over the laryngeal inlet. The primary driver of bolus movement across the pharynx is retraction of the midline tongue base, a movement described as tongue base retraction (TBR). In addition to humans and macaques, a wide variety of common animal models also employ TBR during the pharyngeal stage of swallowing (Table 1).

### Biomechanical Mechanisms of Hyolingual Movement and Lingual Shape Change

Movement and shape change of the tongue are produced by external forces exerted by contraction of extrinsic muscles; reaction forces from the bones, teeth, and food bolus; and through internal forces developed by the intrinsic and extrinsic muscles interweaving within the tongue. Importantly, these internal forces are not just aligned with the long axes of muscle fibers and fascicles. Muscles are isovolumetric, so as they contract in one direction they bulge in orthogonal directions [210]. If bulging is prevented, the ability of the muscle to generate force, strain, and work is affected, so that interactions between muscles and their surrounding connective tissues are crucial for muscle function [212–213]. Indeed, exerting pressure on an active muscle modulates force in a length-dependent manner; increasing intramuscular pressure decreases the ability of a muscle to generate force at short muscle lengths but increases it at long muscle lengths [214].

**Fig. 8:**
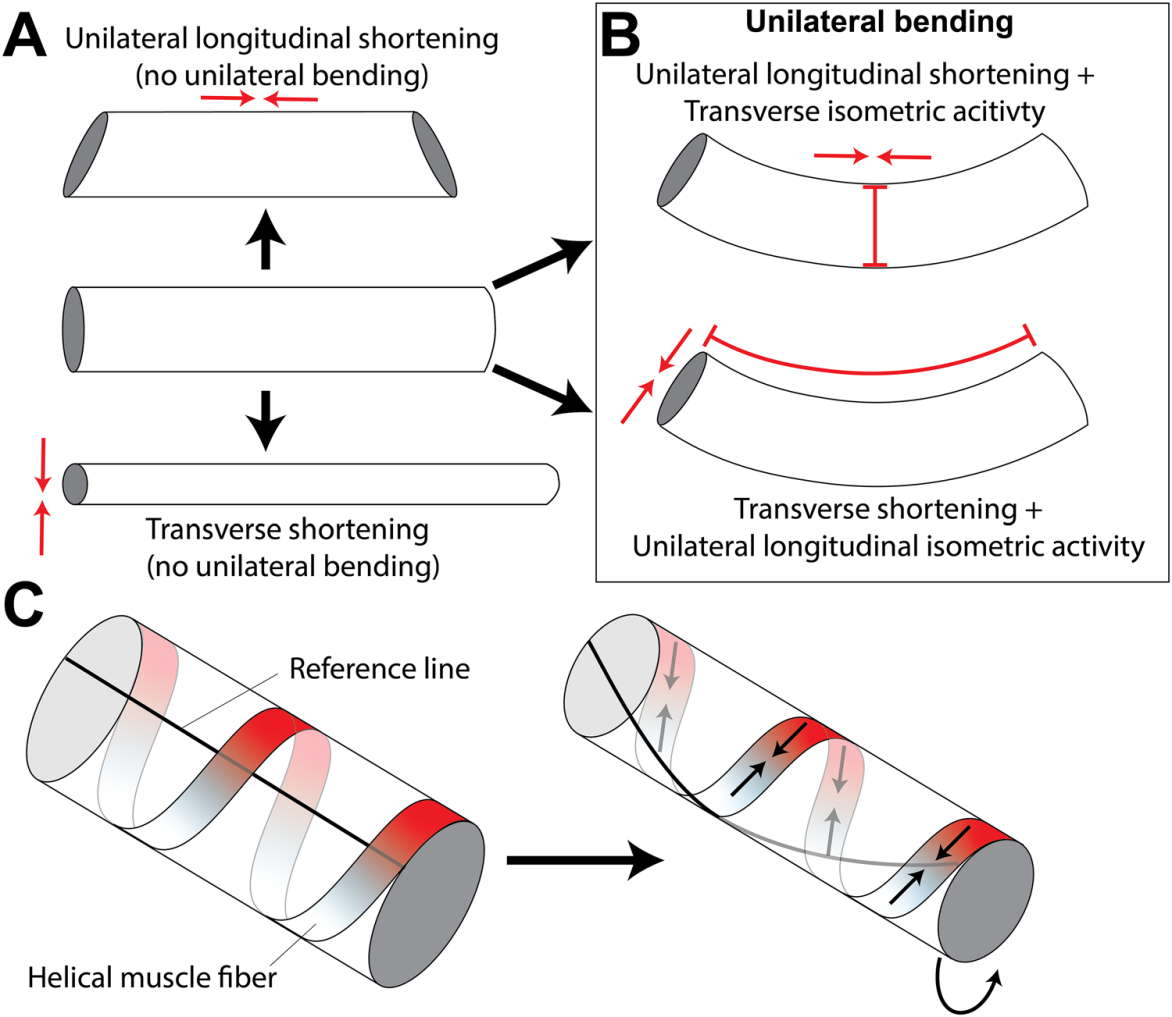
A simple cylinder model shows how activity of different intrinsic muscles can actuate different movement and deformation patterns in a muscular hydrostat. **A** Unilateral shortening of longitudinal fibers with no transverse fiber resistance leads to unilateral bulging (top), while transverse fiber shortening with no longitudinal resistance leads to global lengthening. **B** Unilateral bending can occur through synergistic activity of unilateral shortening of longitudinal fibers with isometric activity of transverse fibers to resist bulging (top), or transverse fiber shortening with unilateral isometric longitudinal activity. **C** helical fibers can cause twisting and lengthening of a muscular hydrostat. Modified fromModified from [215]

This recent work on muscle function confirms the theoretical predictions of the muscular hydrostat model of tongue function [215]. This model posits that, because the tongue is composed principally of skeletal muscle, which in turn is composed principally of fluid, the tongue is essentially isovolumetric. As a result, decreases in one dimension—through muscle shortening—must be accompanied by increases in orthogonal dimensions (Fig. 8). The power of the hydrostat model to explain change in tongue dimensions (e.g., protraction) with reference to muscle shortening in other dimensions (e.g., width and height) is also important for other fleshy organs that have rigid and muscular attachments at one end, but which need to expand away from those attachments and adopt a wide range of shapes, including cephalopod tentacles and elephant trunks.

The theory of muscular hydrostats includes mechanisms for shortening, elongation, bending (flexion) and torsion [215] (Fig. 8). It is intuitive that an isovolumetric structure will shorten if longitudinal muscles contract, but it is perhaps less obvious that circumferential or radial muscles must simultaneously relax. Conversely, a muscular hydrostat will elongate if transverse, circular, or radial muscles contract so as to decrease the cross-sectional area, while any longitudinal muscles are relatively relaxed, akin to relaxation seen in reciprocal inhibition among antagonist pairs of joint-crossing muscles. Theory also predicts that muscular hydrostats can bend (flex) if shortening of longitudinal muscles on one side is coordinated with contraction of transverse or circumferential muscles to prevent increase in cross-sectional area. Simultaneous contraction of both longitudinal and transverse muscles will stiffen the organ, much like isometric contraction will stiffen a muscle. A muscular hydrostat can be twisted using helically arranged muscle fibers such as those seen in tentacles; importantly, these helical fibers can shorten or lengthen the organ while they twist, depending on the orientation of the muscle fibers relative to the long axis (Fig. 8). This fiber arrangement means that in some cases the same helical muscle fibers that are used to twist and lengthen an organ can then twist and shorten it [215], raising the possibility that the same muscles might be recruited for protraction and retraction. Hence, one of the key predictions of muscular hydrostat theory is that muscle synergies are important for producing appropriate movements [216]: to date muscle synergy analyses have not been performed on tongue muscle EMG data. The theory of muscular hydrostats has been applied to explain the biomechanics of squid tentacles, octopus arms, elephant trunks, and vertebrate tongues [216, 217], however, its application to mammal tongues has not been without difficulty. For example, in vivo measurement of human tongue kinematics using tagged MRI are essentially 2D in nature, so that applying the hydrostat model requires the assumption that the tongue is isovolumetric and out-of-plane shear strains are negligible [218, 219]. Studies of primate and pig tongues suggest that, although an assumption of constant volume may be true for the organ as a whole, it is not true regionally [33, 39, 206]; moreover, observed kinematics of the macaque tongue base during swallowing require coronal and transverse shear—shear in coronal and transverse planes—between the core and sides of the tongue [33]. Thus, although the muscular hydrostat model is an important mechanism of tongue motor control, it does not explain all details of tongue kinematics at all scales.

#### Tongue Protraction and Retraction

Tongue protraction and retraction are both fundamental for transport of food through the oral cavity, but tongue protraction is best studied. Genioglossus is the principal driver of whole-tongue protraction, as used in clinical testing of hypoglossal nerve function. Genioglossus-driven tongue protraction is also used by an implantable device that treats sleep apnea by stimulating the medial branch of the hypoglossal nerve to translate the tongue base anteriorly and thus increase the cross-sectional airway of the oropharynx [15]. Tongue protraction can also be produced by hyoid protraction via geniohyoid contraction, and/or by a hydrostatic mechanism involving contraction of transverse and/or vertical intrinsic tongue muscles. How these different mechanisms of tongue protraction are coordinated or selected is unknown.

Studies of pig feeding reveal that the tongue is at similar lengths during drinking and chewing, but during drinking the tongue tip is protruded outside of the oral cavity for most of the gape cycle, protracting and retracting very little, whereas during chewing the tongue protracts and retracts much more [38, 39]. These data suggest that mechanisms of tongue protraction and retraction can be decoupled from mechanisms driving tongue length. Olson et al.’s data also show that different parts of the tongue contract and expand independently. They measured distances between intra-lingual marker pairs in five AP regions of the tongue during chewing and drinking and found that in both behaviors the timing of maximum and minimum overall tongue lengths did not correspond to maxima and minima in their five tongue regions. Some of this temporal incongruence might be due to tongue flexion and twisting, but it seems that overall tongue length is the product of independent lengthening and shortening of different tongue regions [39]. Their data also reveal variability between regions in whether regional length covaries with its width, as predicted for muscular hydrostats. Lacking regional height measures it is difficult to definitively evaluate the muscular hydrostat model using their data. The pig XROMM data were not collected simultaneously with EMG data, but EMG data from other studies confirm that genioglossus is active during tongue-protrusion, as are vertical and transverse intrinsic muscles [39, 202]. Clearly there is still much to learn about control of muscle activity during tongue-protrusion and retraction in both animal models and humans.

#### Flexion and Twisting

The tongues of humans, macaques, and pigs flex and twist during stage 1 transport and chewing cycles in order to position the food bolus between the teeth. How might these movements be produced? Abd-el-Malek hypothesized that twisting of the dorsum of the tongue to the working side is mainly due to unilateral contraction of the contralateral styloglossus [161]. Tomura et al. hypothesized that twisting to the working side is due to contraction of working side hyoglossus, styloglossus, and longitudinal muscles, and of balancing side styloglossus and transverse intrinsic muscles [162]. The theory of muscular hydrostats suggests that symmetrical dorsal or ventral tongue flexion could also be produced by symmetrical contraction of superior or inferior longitudinal muscles accompanied by contraction of transverse or vertically oriented muscles, whereas twisting can be produced by helically arranged fibers [217].

These hypotheses are yet to be tested because of a lack of simultaneous EMG data on muscle activity and 3D data on tongue muscle function during chewing. However, some conclusions can be drawn from the available 3D kinematic data from macaques [36], from non-synchronous biplanar radiographic data from humans [162], and from non-synchronous kinematic and EMG data from pigs [39]. The data from macaques and humans concur that rolling or rotation of the tongue is greater at anterior than posterior sections, confirming that the term “twisting” is the most accurate descriptor of the rotations about AP axes. Macaques and humans both display greater twisting of the tongue during stage 1 transport than during chewing; Tomura et al. further note that in humans maximum tongue twisting occurs around maximum gape during stage 1 transport and early chews, but then shifts to the middle of closing (presumably around the FC/SC transition) in later chews. (Feilich et al.’s data do not control for chew number, but the average timing of peak flexion and roll is just after maximum gape (Fig. 7)). Together these data suggest that bolus size may impact the timing and magnitude of maximum twisting, or that twisting may be important for posterior transport of the bolus during stage 1.

The rotations of vertical and horizontal lines in middle and posterior tongue planes are very similar in magnitude, suggesting that twisting is associated with little if any shear of the tongue *within* these coronal planes (Fig. 7D). Strain data also suggest that there is little shear out of coronal planes—the strain magnitudes between lateral markers in the middle plane and either the tongue tip or the posterior plane are similar on both working and balancing sides (Fig. 7E). The near symmetry of right-left strain data also suggest that lateral tongue flexion is not an important component of tongue kinematics during chewing; instead, shortening of the distance from middle tongue to the tongue tip is simultaneous with, and contributes to, tongue tip retraction and tongue sagittal flexion.

Unfortunately, there are no published data on intrinsic muscle activity recorded simultaneously with 3D tongue movements in macaques or pigs. If we assume that the Supplementary Video from Olson et al. is representative of tongue flexion and twisting during opening in pig chewing cycles, we can use the EMG data published by Kayalioglu et al. (their Fig. 5) to propose some muscular mechanisms [202]. During opening, activity in the inferior longitudinal muscles begins before activity in the superior longitudinals, muscle activity that would result in lingual ventroflexion. Moreover, there is significant working/balancing asymmetry in activity of styloglossus, genioglossus and the inferior longitudinals. During opening the balancing side inferior longitudinal and genioglossus muscles are active before the working side and the working side styloglossus is active before the balancing side. Exactly how these asymmetries might be responsible for tongue twisting in pigs during chewing remains to be determined. In order to answer this question, intrinsic EMG data are needed that are collected simultaneously with 3D intrinsic tongue kinematics.

#### Tongue Base Retraction During Swallowing

**Fig. 9:**
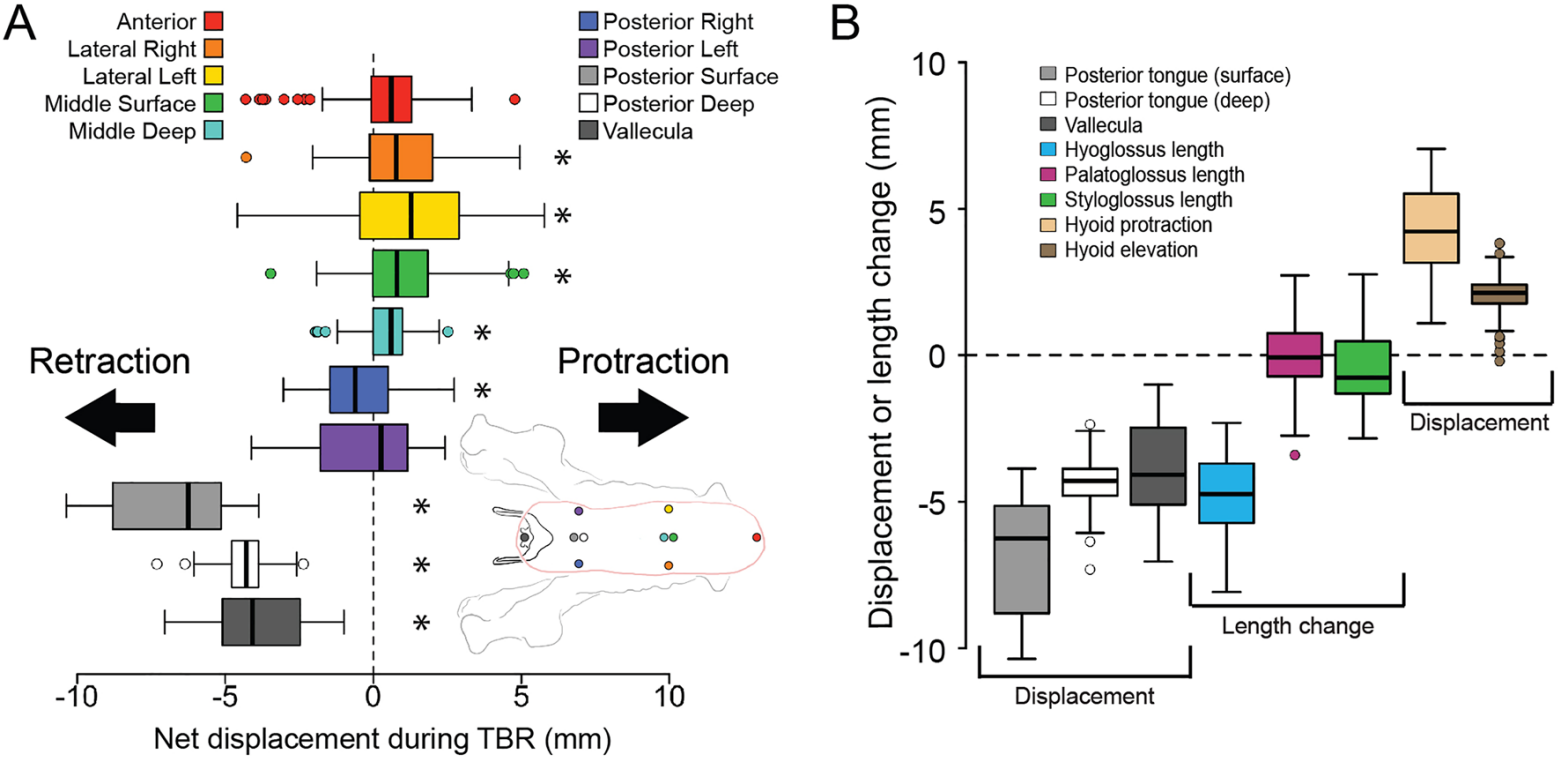
**A** Tongue marker anteroposterior displacement during tongue base retraction (TBR). Measurements were taken relative to the marker’s position at the start of TBR. Positive values indicate protraction; negative values are retraction. Data are averages across four monkeys (two females, two males). Boxes, interquartile range; thick bars, median; error bars, data range; circles are outliers. Asterisks indicate the mean is significantly different from zero using a one-sample Wilcoxon signed rank test. Arrows indicate marker trajectories. Colors of boxes match markers in the accompanying diagram: red, tongue tip, anterior; orange, right lateral; yellow, left lateral; green, middle surface; light blue, middle deep; dark blue, posterior right; purple, posterior left; gray, posterior surface; white, posterior deep; dark gray, vallecula. The pink outline of the tongue indicating marker position relative to the mucosa is based on diceCT data. **B** Mean displacements and extrinsic lingual muscle length changes during tongue base retraction (TBR). Positive values indicate hyoid protraction, hyoid elevation, or muscle lengthening; negative values indicate TBR or muscle shortening. Boxes indicate the interquartile range, thick bars indicate median, error bars indicate data range, circles are outliers. Arrows indicate the trajectory of hyoid movement and muscle shortening. All differences among tongue marker retraction and muscle length change were statistically significant after Bonferroni correction except for the differences between posterior deep and vallecular marker retraction and between posterior deep retraction and hyoglossus shortening. Modified from [33]

The mechanistic driver of TBR during swallowing in humans is debated [33]. One hypothesis posits that contraction of posteriorly directed extrinsic tongue muscles—namely, hyoglossus and styloglossus—pulls the tongue base posteriorly [220, 221]. Although hyoglossus and styloglossus are both active during swallowing in humans and macaques, that does not mean that they are actually shortening: there is ample evidence that hyolingual muscles are not always concentrically active during feeding [33, 64, 222]. Moreover, they attach to the sides of the tongue, not the midline, begging the question of how they might apply a posteriorly directed force to the midline tongue base. Indeed, we have shown that in one macaque the styloglossus was strongly active during TBR but it did not shorten [33] (Fig. 9B). Hyoglossus does shorten during TBR, but this does not retract and depress the lingual insertion; instead it elevates and protracts the hyoid attachment. Moreover, Orsbon et al. show that the midline tongue base retracts *relative to the lateral tongue base* where the hyoglossus and styloglossus insert (Fig. 9A). Together these data suggest that contraction of extrinsic muscles inserting on the sides of the posterior tongue cannot be responsible for TBR in macaques. Moreover, they also suggest that, contrary to assumptions of MRI-based studies [218, 219], there must be shear in transverse planes, between the middle and sides of the tongue, suggesting that any hydrostat properties are modified by intra-lingual connective tissue structures.

A second hypothesis posits that TBR during swallowing is driven by a muscular hydrostat mechanism. Under this model, contraction of transverse intrinsic muscles in the posterior tongue and the tongue base can result in elongation of the tongue base along the path of least resistance, into the oropharynx [218, 219]. The tongue base of macaques gets taller and longer during TBR, but not narrower. Alternative to ML contraction, a muscular hydrostat mechanism of TBR might also be expected to occur via contraction of vertical intrinsic muscles to increase the posteriorly directed component of TBR; however, this would depress the tongue surface and impair the squeeze-back mechanism of swallowing, which requires contact between the tongue surface and the palate. In addition, the assumption of constant volume does not seem to apply to the posterior tongue and tongue base in macaques, both of which actually increase in volume during TBR rather than remaining constant [33].

Instead of relying exclusively on extrinsic muscle shortening or intrinsic muscle contraction of a muscular hydrostat, Orsbon et al. proposed that the primary driving force for TBR in macaques is elevation and protraction of the hyoid bone into the oral volume [33]. This volume, rigidly walled by hard palate, mandible, and teeth, and stabilized inferiorly by a contracting mylohyoid and digastric, cannot accommodate the protracting hyoid, causing the midline tongue base and the food bolus to be squeezed posteriorly. This is a hydraulic mechanism, wherein the hyoid acts as a piston to displace the tongue base posteriorly, with the latter acting like an incompressible fluid in a hydraulic cylinder. Moreover, displacement and possibly force are amplified during a hydraulic-driven TBR: the posterior tongue displaces further and faster than the hyoid protracts, in part because the interface between the inferior tongue and hyoid is larger than that of the posterior oral tongue and tongue base.

A hydraulic mechanism driving TBR during swallowing has so far only been reported in macaques, and inferred for humans [33]. A review of the literature suggests a similar mechanism might apply in other mammals. In hyraxes, tongue kinematics during swallowing is a two phase process: initial bolus movement out of the valleculae occurs without any movement of hyoid or tongue markers, suggesting intrinsic tongue muscle contractions play a role in the first phase of TBR and vallecular emptying [190]. However, the next phase of swallowing, “the tongue/hyoid protraction phase” resembles that of macaques:

“At the beginning of the tongue/hyoid protraction phase of the swallow cycle, the middle tongue marker and the hyoid moved up and forward … but the posterior tongue marker moved up without moving forward. The reduction of anterior movement at this time in a swallow cycle reflects continued posterior movement of the tongue dorsum in the region behind the posterior tongue marker. The changed shape of the bolus showed that the tongue, in conjunction with activity of the pharyngeal constrictors, was continuing to force the bolus towards the oesophagus” (Franks et al. 1985, p. 542). It seems that hyraxes employ both muscular hydrostat and hydraulic mechanisms of TBR during swallowing [190].

Similar movements are described for liquid swallowing in cats [100]: “The posterior part of the tongue … moved backwards as the hyoid continued to move firstly forwards and then upwards (Fig. 2D). At the same time, the tongue became convex dorsally in the transverse axis (as evidenced by a change in the relative positions of lateral and midline markers—Fig. 3F, G). During this time, the bolus passed into the pharynx. The jaws then continued their opening movement, as in a normal lapping cycle, and the hyoid moved forwards (Figs. 1, 2D). Two factors produced the upward-backward sweep of the posterior tongue: movement of the tongue base, i.e., hyoid (Fig. 2D) and change of shape and dimensions within the tongue (Figs. 1, 2F, cf. Fig. 3E with G)” (Thexton and McGarrick [104], p. 336, references to their figures). On p. 338 they continue: “The characteristic upwards/backwards movement of the tongue/hyoid which interrupted the tongue protraction in swallowing cycles (Fig. 2D) was also present in the profiles of tongue marker movement plotted with intra-tongue references (Fig. 2F). The overall movement of the tongue in the swallow period was therefore not just a movement of the tongue base but also a movement within the tongue itself, possibly reflecting styloglossus muscle activity”.

Thus, available data suggest TBR in mammals can be driven by a hydraulic mechanism (macaques and humans) or a mix of hydraulic and hydrostatic mechanisms (hyrax and cats). The diversity of craniofacial and hyolingual anatomy across common animal models hints at the possibility that the mechanisms of TBR may well vary across different species, despite the superficial similarity of the behavior. In humans, compromised TBR performance is commonly observed in dysphagia patients after radiotherapy [223, 224] and stroke [225], and reduced TBR is associated with lower tongue pressure during swallowing and increased post-swallow valleculae residue retention [226]. Understanding the mechanistic diversity of TBR in common lab animal species can better inform animal model selection for studying the etiology and rehabilitation strategy of TBR deficiency among dysphagia patients.

It is important to bear in mind that 2D hyolingual kinematics alone are insufficient to distinguish possible mechanisms of TBR in the absence of other lines of evidence, in particular changes in regional tongue volume and oral volume. Several researchers have found that simple predictions of the muscular hydrostat theory are falsified by regional changes in tongue volume, and data on simultaneous changes in length, width and height. However, if a hydraulic mechanism is acting via the oral volume—oral cavity and the volume above the mylohyoid—this volume must be constricted in order for pressure to build up as the hyoid protracts. Poor swallowing in patients with dentures may reflect lack of control of this oral volume [227]. In order to treat swallowing disorders related to intraoral tongue mechanics, it is critical to understand how hydraulically generated forces interact with lingual muscle and connective tissues to produce changes in regional tongue volumes and tongue pressure exerted on bolus. Detailed studies of intra-lingual anatomy of muscles and connective tissues are clearly needed. How connective tissue morphology relates to shear between adjacent muscular subunits is of particular interest.

If TBR in humans and macaques is driven by hyoid protraction and elevation into the semi-rigid oral volume, what are the muscular drivers of that hyoid displacement? In macaques, simultaneously collected electromyographic (EMG) and XROMM data [33] show that hyoid elevation begins prior to TBR, then combines with hyoid protraction at or just before the onset of TBR. Concentric (shortening) activity in genioglossus often precedes suprahyoid muscle activity, initiating tongue protraction during SC. Genioglossus activity is followed closely by onset of concentric activation of mylohyoid, initiating elevation of the hyoid, then by concentric activity in geniohyoid, which pulls the hyoid forward [33]. The anterior digastric shows two bursts of activity during swallowing, one isometric or eccentric before TBR, coincident with the start of hyoid elevation, and one concentric, peaking after TBR and coincident with rapid jaw depression during FO. [33, 95].

Hyoid movement in humans is sometimes characterized by elevation followed by protraction, but this is not always the case [228]. Hyoid elevation varies with food type and seems to be closely related to events in the oral cavity, whereas hyoid protraction is less variable and associated with opening of the upper esophageal sphincter [229]. Humans also exhibit biphasic activity of anterior digastric and geniohyoid muscles during stage 2 transport and swallow cycles. EMG amplitudes are larger during swallows than stage 2 transport, but two bursts are regularly seen: one during SO when the hyoid is elevated and protracted, and one at the start of FO, when the mandible begins to depress rapidly [208]. There is some evidence that mylohyoid activity begins and peaks before geniohyoid, but the data are not quantitatively presented [230].

In chewing cycles the jaw elevator muscles are usually silent during the SO phase of the gape cycle as the jaw is slowly depressed, as would be expected under the paradigm of reciprocal inhibition. However, during swallow cycles on hard food, both humans and macaques exhibit co-contraction of jaw elevator muscles—masseter, temporalis or medial pterygoid—and jaw depressors—mylohyoid, digastric and geniohyoid—during SO [95, 208, 231, 232]. This co-contraction probably functions to stabilize the mandible while the digastric and geniohyoid muscles elevate and protract the hyoid as the mylohyoid and anterior digastric muscles stiffen the oral volume floor.

### Sensory-Based Modulation of Tongue Movement

The tongue is a truly sensorimotor organ: like the hand, it senses an object while also moving it. The tongue’s primary function is to transport food through the oral cavity, but in the process of transporting food, and in order to transport it effectively, it also collects stereognostic information on the location and physical properties of the food bolus(es). Indeed, the tongue appears to be more important than the palate for oral stereognosis [233]. To perform this function, the mucosa of the tongue is richly innervated with sensory afferents, including diverse nerve endings associated with lingual papillae [234]. The anterior tongue tip is more sensitive than the posterior tongue and soft palate [235–237], and more sensitive than the finger tips for purely tactile sensations—consider the difference in sensitivity between a strand of hair between one’s fingers and the rather annoying sensation of hair in one’s mouth [238]. Greater sensitivity of the tongue tip is important for its role in food manipulation and stage 1 transport, but it is of interest that spatial acuity is low on the posterior tongue and soft palate where the bolus accumulates prior to swallow. We hypothesize that the posterior tongue and soft palate are more sensitive than anterior tongue in proprioceptive and tactile modalities relevant to signaling the swallow-readiness of a food bolus.

#### Lingual Proprioception

Proprioception is the sense of bodily position in space, a sense that emerges from integration of input from multiple kinds of receptors, not just the muscle spindles and Golgi tendon organs traditionally described as mediating proprioception. In the case of the tongue, the sensors on the surfaces of tongue and palate are arguably more important than muscle spindles for lingual proprioception. A simple experiment demonstrates this: when you move your tongue around with your mouth closed you can feel tongue position against the mucosa of the hard palate, but if you open your mouth so wide as to allow your tongue to move without touching lips, palate or teeth, you cannot sense the position of the tongue as it moves. This is probably because muscles spindles are sparse in the tongue tip, but well-documented in the superior and inferior longitudinal muscles, genioglossus, styloglossus, hyoglossus, and transverse and vertical intrinsic muscles [239, 240]. Spindles in transverse intrinsic lingual muscles near the base of the tongue, and in genioglossus, are argued to function in TBR during swallowing and speech [241]. Spindles are also found in human levator and tensor veli palatini, and in the palatoglossus muscles [242, 243], but are sparse in lateral pterygoid [244], digastric, and mylohyoid [245], and completely absent in the superior constrictor muscle of the pharynx [246].

The macaque tongue is also richly endowed with muscle spindles in intrinsic and extrinsic muscles, with Pacinian corpuscles in the midline septum, and with Ruffini endings, a few tendon endings, and some spiral endings resembling those found in extraocular muscles [247, 248]. These afferents leave the tongue on the hypoglossal nerve and enter the CNS on branches of cervical spinal nerves C2–3. Muscle spindles have also been found in the inferior pharyngeal constrictor of macaques [249]. Unlike humans and macaques, rat tongues appear to only have muscle spindles in longitudinal muscles, not in vertical and transverse muscles, and the only extrinsic muscle known to have spindles is the geniohyoid [250]. Careful surveys for muscle spindles in the tongues of rabbits, dogs, and pigs have yet to be performed. Spindles are variably present in the rabbit digastric [251]. It is not known whether these differences from humans affect the utility of these animal models for studies of tongue control.

#### Tactile Sensory Feedback

The importance of effective lingual sensation for chewing and swallowing performance is indicated by data on normal chewing and swallowing function, as well as from experiments where sensory information is blocked. In macaques, during simple transport cycles (lacking a SC phase), tongue protraction begins at or just before minimum gape, and continues until the start of FO, when the tongue starts to retract [84]. When solid food is chewed between the teeth, a SC phase is introduced, the onset of tongue protraction is shifted to before the start of SC, and tongue protraction then continues until the start of FO. Indeed, Hiiemae et al.’s data reveal tight coordination between the timing of tongue tip reversal and the start of FO; tongue tip retraction always occurs within 20–30 ms of the SO/FO transition, so that the duration of SO is related to the distance of tongue protraction. This suggests that the onset of FO is triggered by sensory information from the tongue and/or palate, and that the onset of tongue protraction is triggered by contact of teeth with the food [84]. Some indication of the importance of lingual afferent information for coordinating tongue movements with gape cycle phase transitions comes from studies showing that SO duration is an important driver of gape cycle duration in lizards [252, 253], macaques, and cats [60, 254].

Thexton et al. report similar results in opossums [255]. As in macaques, during the simple transport cycles used for liquid lapping there is no SC phase and tongue retraction changes to protraction just prior to minimum gape. However, when placement of food between the teeth inserts a SC phase, the onset of tongue protraction occurs earlier, around the start of SC. In lapping cycles there is essentially no FO phase because transport of the liquid bolus does not require it, so rapid tongue retraction occurs at a narrow gape. When chewing small pieces of soft food, SC and tongue protraction start at a narrow gape, and when chewing larger pieces of food, SC and tongue protraction are initiated at larger gapes. As in macaques, during transport cycles tongue protraction continues until the end of SO. Thexton et al. hypothesize that the presence of solid food in the oral cavity triggers insertion of a FO phase during which the jaw is rapidly depressed, with the magnitude of the maximum gape varying with the size of the food bolus. These data suggest that, as in macaques, afferent information signaling tooth-food contact also triggers the onset of tongue protraction, whereas afferent information from the tongue and palate modulates jaw kinematics during opening [255]. Thexton et al. noted that the motor pattern of the infra-, anterior and posterior suprahyoid muscles during FO resembles that elicited by the jaw opening reflex, and hypothesized that sensory signals from the tongue indicating the presence of hard food trigger the recruitment of the jaw opening reflex to produce jaw gapes appropriate for transport of solid food. Indeed, they hypothesized that FO and FC—the fast phases of the gape cycle—are added onto the basic lapping cycle in the presence of solid food. Covariation between FO and FC jaw movement velocity suggests that the fast phases may be a module of motor control in cats [254] and in rabbits [256]. These are also the phases during which tongue flexion and roll reposition the food item between the teeth during chewing cycles [36]: how modulation of the durations of the fast phases is linked to modulation of intra-oral tongue kinematics is of interest for future research.

Ingested bolus properties also impact hyoid kinematics. In primates and cats the hyoid begins to move forward relative to the mandible as the jaws start to open [106]. During lapping of liquids, SO is long and there is no FO, so hyoid protraction relative to the mandible is complete and hyoid retraction begins before the end of SO. However, when solid food is introduced into the mouth hyoid protraction persists through the SO/FO transition, so the hyoid is still moving forward relative to the mandible as the mandible rapidly depresses during FO. This anterior hyoid movement may be important for positioning the tongue as it flexes and rolls during FO (Fig. 7). As noted above, in rabbits the posterior tongue is still protracting while anterior and middle tongue are retracting at the same time as the tongue is flexed [169]. These data suggest that hyoid position is coordinated with 3D intra-oral tongue kinematics during chewing in mammals, including humans.

#### Peripheral Sensory Disruption

Peripheral perturbations—nerve blocks or transections—provide insight into the specific role of information from sensory afferents in feeding performance. Oral sensation is crucial for all stages of feeding, including its initiation [257, 258]. Bilateral trigeminal sensory nerve transections in rabbits extend the duration of feeding sequences and introduce variability into the pattern of jaw movements, probably due to incoordination of the tongue and lips during mastication [259]. Similarly, unilateral nerve block of oral sensory afferents in pigs reduces the ability to modulate jaw movement and bite force in response to different foods, resulting in “clumsy” food handling [260]. Unable to visualize lingual motions, these authors could only speculate about the changes to tongue kinematics that might underlie this decrease in performance.

**Fig. 10:**
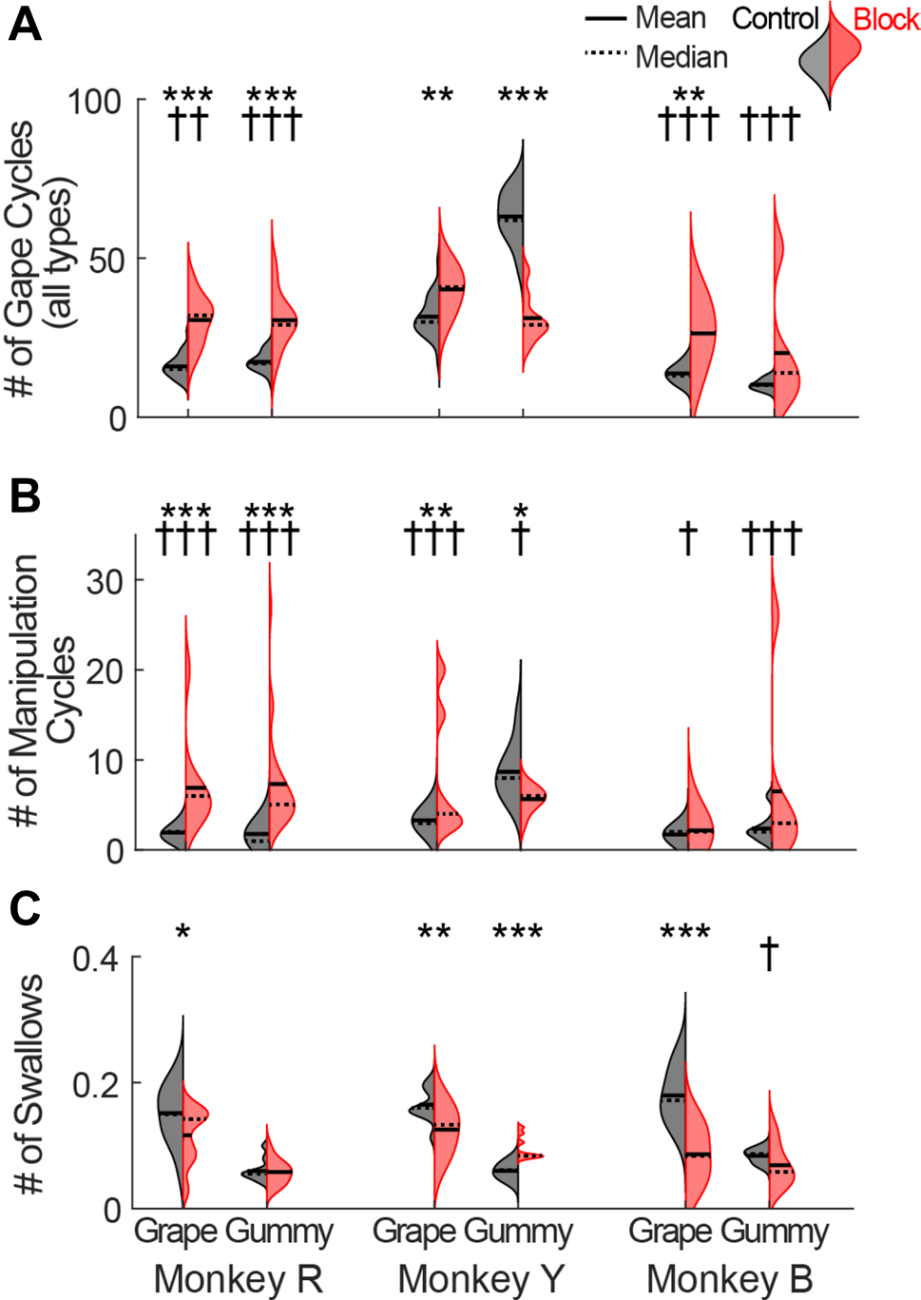
Effects of nerve block on feeding performance variables for three monkeys eating two food types. Left halves of hemi-violins (black) are control and right halves (red) are nerve block for a single food type for an individual. **A** Total number of gape cycles (all cycle types) per food item, from initial ingestion of food to terminal swallow. **B** Number of gape cycles, manipulation and/or stage I transport, prior to the onset of rhythmic chewing. **C** Swallow frequency, as measured by number of swallows per 10 gape cycles. Results of a two-tailed t-test and F-test of equality of variances (within each subject) are indicated by asterisks and crosses, respectively: *^,†^*P* < 0.05; **^,††^*P* < 0.01; ***^,†††^*P* < 0.001. Horizontal solid lines are means and horizontal dashed lines are medians. Modified from [35]

**Fig. 11:**
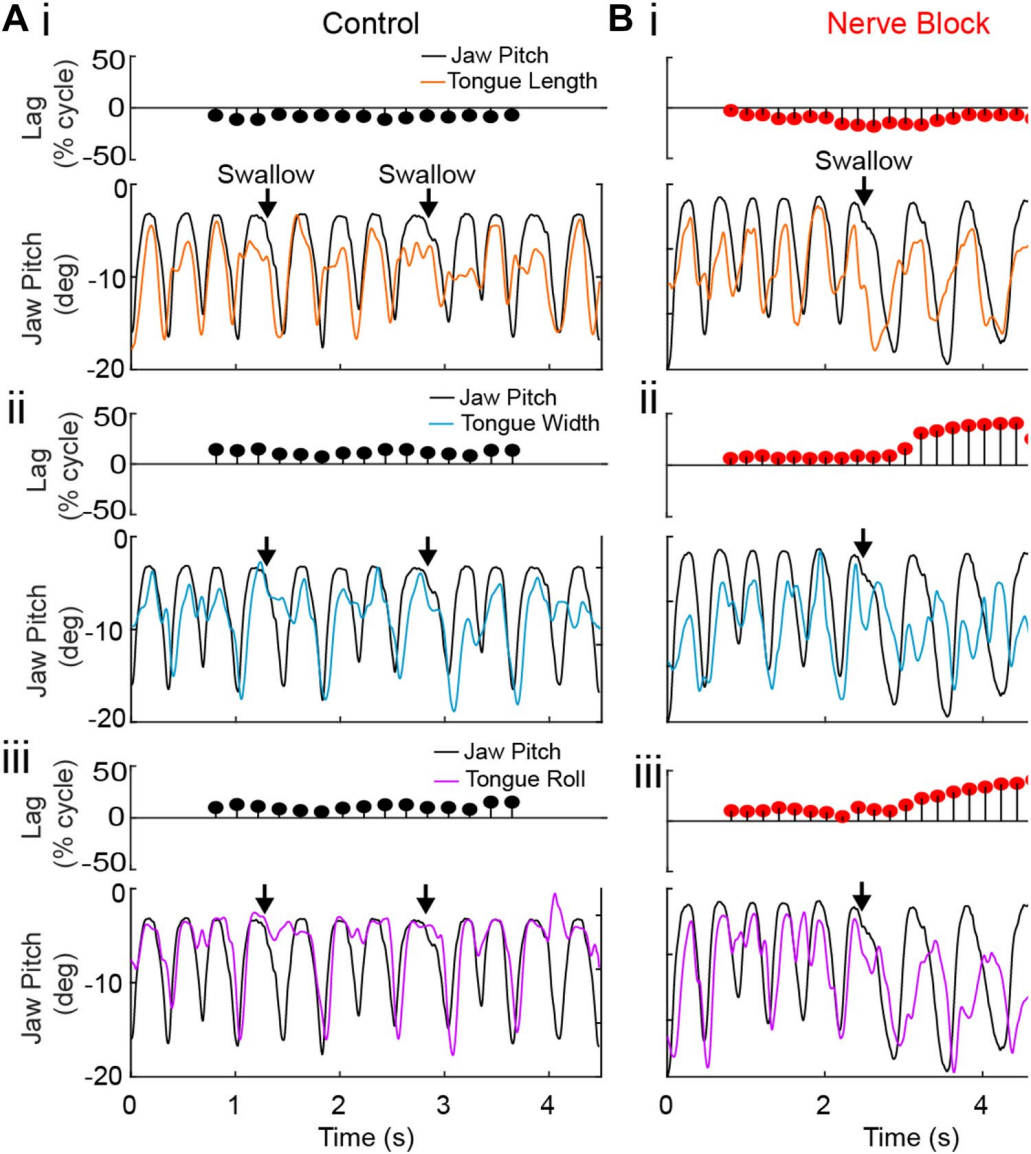
Representative kinematic traces of tongue movement relative to jaw pitch in control (**A**, black) and trigeminal nerve block (**B**, red) conditions. The upper ball-and-stick plots depict the lag of jaw pitch (black lines) and anterior tongue length (i, orange), width (ii, teal), and roll (iii, pink). Lags correspond to the maximum of the cross-correlation function of the two signals for a 300-frame range centered at that position. Note that in (**A**), the correlation between jaw pitch and tongue width remains temporally consistent over the course of 10 chews and two intercalated swallows. In (**B**), the initial lags are similar to (**A**), but then shift substantially at ~ 2.5 s. Swallows are indicated by black arrows

Recent use of XROMM to quantify 3D jaw and tongue movements provides further insight into effects of sensory perturbations in pigs and macaques. Unilateral lingual nerve transection in pigs impacts both mandibular kinematics and tongue–jaw temporal coordination, but there were high amounts of inter-individual variability in the response to sensorimotor perturbation [192, 261]. Bilateral nerve block of trigeminal sensory afferents from the oral cavity in macaques decreases feeding performance, again with inter-individual and food effects (Fig. 10) [35]. For example, the number of gape cycles in a feeding sequence increased in all three animals when eating grapes, and in two animals when eating gummies, whereas one animal chewed gummies for fewer cycles after the nerve block. Similarly, the frequency of intercalated swallows increased for grape chewing in all three animals, but during gummy chewing the number of swallows increased in one animal and decreased in another. Chew cycle durations mostly increased, and this was mostly due to an increase in FO and, less so, in FC. Given that the fast phases are when the tongue is flexing and rolling to reposition the food bolus, we were interested in the effects of the nerve block on 3D tongue kinematics. Interestingly, average kinematic trajectories—roll, length and width—were relatively unaffected by sensory loss, but became more variable, as did their temporal correlation with jaw movement (Fig. 11). As in pigs [261], the effects on coordination are also reflected in alteration and increased variation in the relative timing of tongue tip reversal from protraction to retraction. These results confirm that during mastication oral sensory afferents are important for maintaining consistent and efficient patterns of tongue-jaw coordination—relative timing—in the face of changing bolus properties, but may be less important for generating the motor patterns underlying lingual shape change.

The trigeminal nerve block used in our lab does not block afferent signals from lingual muscle spindles that return to cervical spinal cord levels C2 and C3 via the hypoglossal nerve [247]. Human data suggest that these proprioceptive afferents may provide information on bolus size and position that allows chewing and swallowing to continue, albeit less efficiently [262]. Topical anesthesia of the tongue and palate in humans reduces spatial sensitivity but has no effect on the ability to perceive the size of objects in the mouth. There was no relationship between spatial sensitivity and the ability to perceive object size, nor with the median particle size at different time points, nor on chewing time, or number of chew cycles before swallowing. These data suggest that information from lingual muscle spindles may be sufficient for detection of bolus volume, which is important for modulating hyoid kinematics prior to swallow onset [183].

In sum, these data from animal models corroborate human studies showing that tongue sensory function impacts feeding performance [263, 264], that lingual sensation may be more important than palatal sensation [265], and that oral sensory loss affects tongue-jaw coordination and intra-oral tongue kinematics. These results open new avenues of research into how swallowing performance in human patients may be affected by loss of afferent signals from the tongue following lingual nerve damage, insensate tongue reconstruction, or restriction of afferent signals through prescription of homogenously textured diets.

### Role of Cortex in Swallowing and Tongue Movements

#### Human Studies

While much of the direct neural control of feeding-related tongue movements resides in subcortical structures, particularly the motor nuclei of the brain stem, the cortex modulates and regulates these movements on a routine basis, especially when they are voluntarily initiated and controlled. Early surface electrical stimulation studies in humans identified an area on the precentral gyrus lateral to the orofacial motor cortex (M1o) and close to the Sylvian fissure that evoked swallowing behavior when stimulated [266, 267]. More recently, subthreshold transcranial magnetic stimulation in humans was able to map out distinct zones on the lateral precentral gyrus that evoked muscle activity in three muscle groups involved in swallowing [268]. In particular, activity in the mylohyoid muscle involved in lifting the hyoid bone and tongue was evoked by stimulating more anterior-laterally on the precentral gyrus than the sites that evoked activity in the pharyngeal muscles, which in turn were more anterior-lateral to the sites where activity in esophageal muscles was evoked. This study also reported some asymmetric effects between the two cerebral hemispheres, effects that were variable across subjects and not linked to subject handedness.

The advent of functional magnetic resonance imaging (fMRI) in the 1990s made it possible to map out human cortical activity associated with automatic and voluntary swallowing. Cortical activation associated with swallowing has been found in lateral pre- (M1o) and post-central (orofacial somatosensory cortex, S1o) gyri, fronto-parietal operculum, insula, anterior cingulate cortex (ACC), supplementary motor cortex (SMA), cuneus and precuneus [271–275]. Although each study found slightly different results, likely due to variations in behavioral paradigm, they consistently found that both voluntary and automatic swallowing were associated with activity within the peri-central cortex bilaterally and insular cortex predominantly on the right side [269, 270, 272, 273]. Direct comparisons revealed stronger activity in anterior cingulate cortex activity during voluntary, instructed swallows versus naïve—non-instructed—saliva swallows [271]. Besides the right-sided asymmetry of insula activation, a number of studies have also observed a left-sided bias in the peri-central cortex during certain swallowing conditions [272–273]. More specifically, a recent magnetoencephalography study found stronger left-sided laterality for voluntary versus automatic, reflexive swallowing [276]. This left-sided bias is noteworthy because it is known that left-sided stroke results in swallowing apraxias (see *Disruptions in Cortical Function* section below) associated with the more voluntary oral phase of swallowing [277].

Although stimulation studies have identified a swallow-triggering zone lateral to orofacial sensorimotor cortex, fMRI studies suggest activation beyond the orofacial sensorimotor cortex involved in controlling tongue and other orofacial structures. Indeed, by directly comparing activation during voluntary swallowing and voluntary tongue elevation tasks in the same subjects, a large overlap of activation was found for both tasks within the orofacial sensorimotor cortex as well as the fronto-parietal operculum, SMA, and ACC, although total activation volume was larger for tongue elevation versus swallowing [272]. Therefore, based on cortical activation studies in humans, it is not clear that there is a dedicated cortical zone for swallowing; rather, it appears that swallowing shares similar orofacial neural representations with other movements of the tongue.

#### Cortical Function in Control of Tongue Function During Chewing and Swallowing in Non-human Primates

Three decades of neuronal recordings, intracortical microstimulation (ICMS), and receptive field mapping in nonhuman primates suggest that orofacial motor cortex (M1o), orofacial somatosensory cortex (S1o), and the cortical masticatory area (CMA) play partially distinct but overlapping roles in control of orofacial behaviors including mastication, tongue function, and swallowing [48, 278–282]. All three areas are active throughout the feeding sequence and receive afferent inputs from bilateral orofacial structures [283–286]. In M1o, standard short duration ICMS protocols (35 ms duration, 333 Hz, < 30 μA) evoked twitch-like movements of the face, jaw, and tongue but not swallows [287]. As is seen in upper-limb motor cortex [288], face, jaw, and tongue sites were partially overlapping such that a single site evoked movement in more than one body part. Moreover, multiple non-contiguous zones evoked the same movements [289]. Nevertheless, there was evidence of a crude somatotopic organization where facial sites formed a horseshoe pattern caudally, rostrally, and medially surrounding jaw and tongue sites that were generally located more laterally. On the other hand, longer stimulation trains of 3 to 4 s in duration at 50 Hz resulted in swallowing in M1o as well as S1o, CMA, and deep to CMA in white matter and frontal operculum [290]. Although there were sites that evoked only swallows, particularly in and deep to CMA, most sites that evoked swallows also concurrently evoked other orofacial movements, including mastication. Interestingly, the temporal sequence of muscle activations was similar for ICMS-triggered swallows and natural swallows, such that the genioglossus and masseter muscles activated nearly synchronously followed by activation of the cricothyroid muscle [290].

Electrophysiological recording studies have examined modulatory responses in MIo, SIo, and CMA during trained tongue movement tasks [291–297]. As the tongue is intimately involved in the oral stage of food processing and the pharyngeal stage of swallowing in particular, understanding how neurons in these areas modulate with voluntary tongue movements may provide insights in their role during swallowing. Tuning of tongue direction in a trained tongue-protrusion task was observed in single cells of M1o and S1o [291, 295]. More recently, we have used multi-electrode arrays to examine population coding of tongue direction as monkeys performed isometric force protrusions in three different directions: forward, leftward, and rightward [297]. We found that simultaneously recorded populations of M1o and S1o (population size of 60–80 units per area) could reliably decode single-trial tongue direction with accuracies up to 90% (chance at 33%). While both areas showed comparable performance, the temporal evolution of decoding differed across areas. M1o decoding performance exhibited a late-onset and abrupt increase relative to force onset followed by transient performance that decreased prior to force offset. In contrast, S1o population decoding improved earlier with a slower ramp up followed by sustained performance until force offset. A similar trained tongue-protrusion task was performed with single-unit recordings in M1o but modulation was examined not only during the tongue task but also during swallows [296]. By identifying tongue-related sites using short-train ICMS, it was found that a majority of these tongue sites recorded neurons that modulated their activity with swallows. Three types of phasic modulation were observed, occurring either before, during, or before *and* during swallowing.

#### Disruptions in Cortical Function

Naturally occurring or experimentally induced cortical perturbations provide additional insight into the role of the cortex in swallowing and tongue function. Over half of ischemic strokes occur in the region of the middle cerebral artery which supplies OSMcx [298], and unilateral strokes of human sensorimotor cortex are usually associated with loss of chewing and swallowing function. Up to 78% of stroke survivors experience dysphagia acutely and 11–50% of patients have dysphagia after six months with resulting mortality and morbidities, including dehydration, malnutrition, aspiration pneumonitis, and pneumonia, tracheostomy, gastric tube placement, and diminished quality of life [298–304].

In human stroke patients with unilateral ischemic cerebrovascular accidents either on the left or right side, videofluoroscopy revealed lateralized dysfunction of different phases of swallowing [277]. In particular, patients with right-sided stroke experienced pharyngeal dysphagia, as evidenced by pharyngeal pooling of the bolus and aspiration. In contrast, left-sided stroke patients had swallowing difficulties affecting the oral phase, based on longer mean pre-pharyngeal response times, as well as oral apraxias, as evidenced by a lack of coordination of labial, lingual, and mandibular movements. Interestingly, this left-sided bias for oral apraxias is consistent with similar lateralization of cortical damage associate with upper-limb apraxias [305, 306]. A follow-up study by the same research group examining unilateral ischemic middle cerebral artery stroke patients also found longer pharyngeal response durations and more frequent aspiration for right-sided as compared to left-sided strokes, although both groups exhibited longer total swallowing durations compared to controls [307]. Also, consistent with fMRI studies, a small videofluoroscopic study revealed that three patients with isolated lesions of the anterior insular cortex due to stroke exhibited delayed initiation of pharyngeal swallowing [308].

Experimentally induced cortical lesions in non-human primates provide more refined localization of orofacial function than usually observed with stroke. Similar to humans, irreversible unilateral lesions encompassing M1o and S1o in macaques resulted in crossed hemi-paresis or -paralysis of the lower facial muscles: i.e., weakness or paralysis of the muscles of the lips and cheeks on the side of the face contralateral to the lesion [309]. Qualitative observations revealed that the tongue was also weakened, and at rest the distal third of the tongue was curved toward the side of the lesion, except for the tongue tip, which curves back towards the contralesional side. This posture is remarkably similar to the tongue shape transiently seen during the fast open phase of chewing cycles [36]. Recovery of function after unilateral lesion of M1o was limited, even after 5 weeks. Using cold block to reversibly disrupt cortical function in S1o unilaterally resulted in only very limited effects on the performance on a trained tongue task and chewing, and no effect on swallowing was found [49]. Bilateral, but not unilateral, M1o ablations resulted in difficulty controlling jaw movements during a trained bite-task [79]. How disruption of cortical activity impacts 3D intraoral tongue movements remains to be quantified.

Bilateral lesions of M1o and CMA resulted in bilateral paralysis or paresis of muscles in the lips and cheeks (innervated by cranial nerve (CN) VII) and in the distal two-thirds of the tongue (CN XII), impacting ingestion and food manipulation, and problems with pushing food to the back of the mouth (i.e., the oral phase of swallowing), but had little if any effects on jaw muscles (CN V_3_), with no obvious effects on chewing and pharyngeal phases of swallowing. Recovery of function after bilateral M1o lesions was minimal, even after 8 weeks [77, 309, 310]. Similarly, reversible bilateral cold block of M1o and S1o primarily impaired food manipulation/stage 1 transport and increased the pre-swallow duration but not swallowing [45, 311, 312]. However, reversible bilateral cold block of CMA including lateral M1o resulted in a decrease of the rate of swallowing by a factor of two, an increase in pre-swallow and swallow durations, as well as a concomitant increase in EMG duration and decrease in EMG amplitude in the masseter, anterior digastric, and thyrohyoid muscles during swallowing [313, 314]. Moreover, the relative onset timing of these muscles was altered with cold block.

After temporary inactivation of either M1o [47] or S1o [45], monkeys’ performance on a biting task was largely unaffected, whereas performance of a tongue-protrusion task suffered. This result extended to naturalistic behavior as well; Lin et al. found that temporary S1o inactivation impaired chewing and swallowing principally through the introduction of tongue motor deficits [311]. Without coordinated tongue movements, animals struggled to transport food to and from the occlusal table during stage I and stage II transport, respectively. Taken together with cortical perturbation studies in non-primates [259, 315], there is strong support for the orofacial sensorimotor cortex’s central role in generating the efficient, coordinated tongue movements and deformations that underlie both mastication and deglutition. Certainly neural activity in OSMcx is closely related to jaw and tongue kinematics. At the population level the neural dynamics of rhythmic chewing vary at multiple timescales, from complete feeding sequences, through feeding sequence stages, to individual gape cycle phases [61]. Most recently, we showed that during feeding in rhesus macaques intraoral tongue kinematics measured using XROMM could be accurately decoded from OSMcx population responses with decoders similar to those used in the context of the upper-limb. In particular, we found that the best decoding performance was achieved from M1o as opposed to SIo [40]. In summary, these data demonstrate how the orofacial sensorimotor cortex plays an important role in control of tongue movement during simple voluntary tasks, such as tongue-protrusion, as well as more complex changes in 3D tongue shape during feeding.

### Areas for Future Work

“Animal models are uniquely able to facilitate understanding and testing of the causal mechanisms that produce good or bad performance in a normal, intact, and healthy individual” [37]. As such, animal models are invaluable tools for testing hypotheses about neural, physiological, and biomechanical mechanisms of hyolingual function and they will continue to be used for chewing and swallowing research for the foreseeable future. However, our review suggests that mechanisms of tongue movement may or may not be the same in different animal models, suggesting that better understanding of tongue function in animal models is needed to fully understand their benefits and limitations as models for human swallowing. Several areas deserve particular attention.

The animal models commonly used for chewing and swallowing research—macaques, pigs, cats, rats, rabbits—vary in the shape, position, and completeness of the bones of the hyoid apparatus. The evolution of this variation presumably reflects selection for performance of several functions under variable conditions, including feeding, respiration, and vocalization. The extent to which this morphological diversity in hyoid shape and posture/craniomandibular position is related to variation in soft tissue anatomy and hyolingual mechanics during swallowing remains to be determined. For example, the basihyal of cats is rod-shaped, similar to humans, but unlike humans is situated well behind the mandible and is connected to the cranium by a continuous chain of small bones. The basihyal of rats is also human-like in shape and in being connected to the cranium by ligaments, but it is located near the back of the mandible at rest. Are mechanisms of tongue base retraction during swallowing in this species the same as in macaques and humans? Published data suggest that in cats TBR during swallowing may be achieved via a combination of muscular hydrostat and hydraulic mechanisms, but in macaques TBR is produced mainly by a hydraulic mechanism [33, 99, 190].

Animal models also exhibit diversity in positional relationships of hyoid bone and thyroid cartilage at rest. Rabbits and cats resemble humans in having a larynx situated below the hyoid, whereas macaques and rats have a larynx positioned behind or just below the basihyal (Fig. 2). Given that thyroid cartilage movement relative to the hyoid is supposed to be an important driver of epliglottal flipping during swallowing in humans, what drives this epiglottal movement in various animal models, particularly in those in which there is little relative movement between the hyoid and thyroid cartilage? Are biomechanical mechanisms of airway protection the same across animal models and humans, and if not, why? We clearly need better 3D measurements of tongue, hyoid and larynx kinematics during swallowing in animal models other than macaques to better understand the diversity or conservation of hyolingual mechanics during swallows. To link these kinematics to muscle activity and morphology we will also need better data on hyolingual muscle morphology in animal models; the high spatial resolution and contrast provided by the diceCT workflow provides the methods for doing this in high resolution [33, 31].

The available data suggest that mammals that chew unilaterally with a medial component to jaw movement during the power stroke—humans, macaques, rabbits, pigs—all twist and flex the tongue to place the food bolus on the chewing side toothrow. However, it is not known whether this shape change and movement is produced using the same muscles in all these animals. This is in part because we lack detailed anatomical data on intrinsic and extrinsic tongue muscles of these animals, but also because 3D data on internal and external tongue kinematics are not available for all these animals. Nor do we know whether similar asymmetrical tongue movements are used by rats, which chew using a bilateral posterior-to-anterior power stroke rather than the unilateral lateral-to-medial power stroke seen in primates. The answer to this question might impact the utility of rats as models for studying the control of intra-oral tongue kinematics during chewing in humans.

A wide range of data from humans and animal models confirms the importance of lingual tactile afferents for coordination of tongue and jaw movements, including relative timing of tongue tip reversal during opening, and even the very presence of a FO + FC motor complex during the gape cycle. Nerve block studies isolating the role of tactile versus muscle spindles in intra-oral proprioception suggest that intra-lingual muscle spindles play an important role in sensing bolus size, which can be used to modulate the oropharyngeal swallow. The apparent similarity in sensorimotor mechanisms of tongue-jaw coordination across animal models argues for their continued use in studies of sensory deficits of lingual function [192, 261], but better data are needed on the distribution of muscle spindles and other proprioceptors in tongues of animal models.

Orofacial sensorimotor cortex (OSMCx) plays an important role in coordinating jaw and tongue movements, and hence maintaining feeding performance. Studies of cortical control of lingual function in macaques are particularly important, given their similarities to humans. These studies are necessary because we know little about how to rehabilitate chewing and swallowing after cortical strokes. This is partly because we know little about acute and chronic effects of unilateral lesions of OSMcx on feeding system function, and we know almost nothing about the roles of bi-hemispheric neuroplasticity and vicariation in OSMcx and orofacial supplementary motor areas in recovery of feeding performance. In order to design brain-machine interfaces or non-invasive cortical stimulation protocols to recover hyolingual function, we need a better understanding of how the cortex controls jaw and tongue movements during feeding. To the extent that cortical involvement in lingual control may be ancestral for the mammalian clade, studies of cortical control of hyolingual function could profitably be performed in non-primate mammals [317].

## References

[1] Ali SO, Thomassen M, Schulz GM, Hosey LA, Varga M, Ludlow CL, Braun AR (2006). Alterations in CNS activity induced by botulinum toxin treatment in spasmodic dysphonia: an (H2OPET)-O-15 study. J Speech Lang Hear Res.

[2] Simonyan K, Ludlow CL (2010). Abnormal activation of the primary somatosensory cortex in spasmodic dysphonia: an fMRI study. Cereb Cortex.

[3] Khedr EM, Abo-Elfetoh N (2010). Therapeutic role of rTMS on recovery of dysphagia in patients with lateral medullary syndrome and brainstem infarction. J Neurol Neurosurg Psychiatry.

[4] Martin RE (2009). Neuroplasticity and swallowing. Dysphagia.

[5] Hamdy S, Aziz Q, Rothwell JC, Power M, Singh KD, Nicholson DA, Tallis RC, Thompson DG (1998). Recovery of swallowing after dysphagic stroke relates to functional reorganization in the intact motor cortex. Gastroenterology.

[6] Hamdy S, Gow D, Hobson A, Furlong P, Rothwell J, Thompson D. Mechanisms of modulation of human swallowing motor cortex following pharyngeal stimulation. In: Digestive disease week and the 104th annual meeting of the American Gastroenterological Association, vol 124. 2003; p 255

[7] Mistry S, Michou E, Hamdy S, Sci MAH (2011). Enhancing the human swallowing motor system by the application of a novel brain stimulation intervention, intermittent theta burst stimulation. Gut.

[8] Mistry S, Michou E, Hamdy S. Application of a novel brain stimulation intervention, intermittent theta burst stimulation to enhance the human swallowing motor system. In: Digestive disease week and the 104th annual meeting of the American Gastroenterological Association, vol 140. 2011; pp. S362.

[9] Jayasekeran V, Rothwell J, Hamdy S (2011). Non-invasive magnetic stimulation of the human cerebellum facilitates cortico-bulbar projections in the swallowing motor system. Neurogastroenterol Motil.

[10] Michou E, Mistry S, Jefferson S, Singh S, Hamdy S (2010). A preliminary study of neurostimulation based interventions in the treatment of chronic dysphagia post-stroke. Gut.

[11] Murdoch BE, Ng ML, Barwood CHS (2011). Treatment of articulatory dysfunction in Parkinson’s disease using repetitive transcranial magnetic stimulation. Eur J Neurol.

[12] Dias AE, Barbosa ER, Coracini K, Maia F, Marcolin MA, Fregni F (2006). Effects of repetitive transcranial magnetic stimulation on voice and speech in Parkinson's disease. Acta Neurol Scand.

[13] Nguyen JP, Nizard J, Keravel Y, Lefaucheur JP (2011). Invasive brain stimulation for the treatment of neuropathic pain. Nat Rev Neurol.

[14] Gustin SM, Peck CC, Cheney LB, Macey PM, Murray GM, Henderson LA (2012). Pain and plasticity: is chronic pain always associated with somatosensory cortex activity and reorganization?. J Neurosci.

[15] Heiser C, Knopf A, Bas M, Gahleitner C, Hofauer B (2017). Selective upper airway stimulation for obstructive sleep apnea: a single center clinical experience. Eur Arch Otorhinolaryngol.

[16] Lambeth C, Amatoury J, Wang Z, Foster S, Amis T, Kairaitis K (2017). Velopharyngeal mucosal surface topography in healthy subjects and subjects with obstructive sleep apnea. J Appl Physiol.

[17] Bogousslavsky J, Van Melle G, Regli F (1988). The Lausanne stroke registry: analysis of 1,000 consecutive patients with first stroke. Stroke.

[18] Schimmel M, Leemann B, Christou P, Kiliaridis S, Herrmann FR, Muller F (2011). Quantitative assessment of facial muscle impairment in patients with hemispheric stroke. J Oral Rehabil.

[19] Schimmel M, Leemann B, Schnider A, Herrmann FR, Kiliaridis S, Muller F (2013). Changes in oro-facial function and hand-grip strength during a 2-year observation period after stroke. Clin Oral Investig.

[20] Schimmel M, Ono T, Lam OLT, Muller F (2017). Oro-facial impairment in stroke patients. J Oral Rehabil.

[21] Barer DH (1989). The natural history and functional consequences of dysphagia after hemispheric stroke. J Neurol Neurosurg Psychiatry.

[22] Han DS, Chang YC, Lu CH, Wang TG (2005). Comparison of disordered swallowing patterns in patients with recurrent cortical/subcortical stroke and first-time brainstem stroke. J Rehabil Med.

[23] Theurer J, Johnston JL, Taves DH, Bach D, Hachinski V, Martin RE (2008). Swallowing after right hemisphere stroke: oral versus pharyngeal deficits. Can J Speech-Lang Pathol Audiol.

[24] Theurer JA, Johnston J, Martin RE (2006). Swallowing after hemispheric stroke: oral versus pharyngeal stage abnormalities. Dysphagia.

[25] Konaka K, Kondo J, Hirota N, Tamine K, Hori K, Ono T, Maeda Y, Sakoda S, Naritomi H (2010). Relationship between tongue pressure and dysphagia in stroke patients. Eur Neurol.

[26] Hori K, Ono T, Iwata H, Nokubi T, Kumakura I (2005). Tongue pressure against hard palate during swallowing in post-stroke patients. Gerodontology.

[27] Malagelada JR, Bazzoli F, Boeckxstaens G, De Looze D, Fried M, Kahrilas P, Lindberg G, Malfertheiner P, Salis G, Sharma P, Sifrim D, Vakil N, Le Mair A (2015). World gastroenterology organisation global guidelines: dysphagia—global guidelines and cascades update September 2014. J Clin Gastroenterol.

[28] Manikantan K, Khode S, Sayed SI, Roe J, Nutting CM, Rhys-Evans P, Harrington KJ, Kazi R (2009). Dysphagia in head and neck cancer. Cancer Treat Rev.

[29] Krebbers I, Simon SR, Pilz W, Kremer B, Winkens B, Baijens LWJ (2021). Patients with head-and-neck cancer: dysphagia and affective symptoms. Folia Phoniatr Logop.

[30] Rogers SN, Heseltine N, Flexen J, Winstanley HR, Cole-Hawkins H, Kanatas A (2016). Structured review of papers reporting specific functions in patients with cancer of the head and neck: 2006–2013. Br J Oral Maxillofac Surg.

[31] Hiiemae KM, Palmer JB (2003). Tongue movements in feeding and speech. Crit Rev Oral Biol Med.

[32] Brainerd EL, Baier DB, Gatesy SM, Hedrick TL, Metzger KA, Gilbert SL, Crisco JJ (2010). X-ray reconstruction of moving morphology (XROMM): precision, accuracy and applications in comparative biomechanics research. J Exp Zool A Ecol Genet Physiol.

[33] Orsbon CP, Gidmark NJ, Gao T, Ross CF (2020). XROMM and diceCT reveal a hydraulic mechanism of tongue base retraction in swallowing. Sci Rep.

[34] Orsbon CP, Gidmark NJ, Ross CF (2018). Dynamic musculoskeletal functional morphology: integrating diceCT and XROMM. Anat Rec (Hoboken).

[35] Laurence-Chasen JD, Arce-McShane F, Hatsopoulos N, Ross CF (2022). Loss of oral sensation impairs feeding performance and consistency of tongue–jaw coordination. J Oral Rehabil..

[36] Feilich KL, Laurence-Chasen JD, Orsbon C, Gidmark NJ, Ross CF (2021). Twist and chew: three-dimensional tongue kinematics during chewing in macaque primates. Biol Lett.

[37] German RZ, Crompton AW, Gould FDH, Thexton AJ (2017). Animal models for dysphagia studies: what have we learnt so far?. Dysphagia.

[38] Olson RA, Montuelle SJ, Chadwell BA, Curtis H, Williams SH (2021). Jaw kinematics and tongue protraction-retraction during chewing and drinking in the pig. J Exp Biol.

[39] Olson RA, Montuelle SJ, Curtis H, Williams SH (2021). Regional tongue deformations during chewing and drinking in the pig. Integr Org Biol.

[40] Laurence-Chasen JD, Ross CF, Arce-McShane FI, Hatsopoulos NG (2023). Robust cortical encoding of 3D tongue shape during feeding in macaques. Nat Commun.

[41] Miller AJ (2002). Oral and pharyngeal reflexes in the mammalian nervous system: their diverse range in complexity and the pivotal role of the tongue. Crit Rev Oral Biol Med.

[42] Fregosi RF, Ludlow CL (2014). Activation of upper airway muscles during breathing and swallowing. J Appl Physiol.

[43] Sawczuk A, Mosier KM (2001). Neural control of tongue movement with respect to respiration and swallowing. Crit Rev Oral Biol Med.

[44] Lowe AA (1980). The neural regulation of tongue movements. Prog Neurobiol.

[45] Lin LD, Murray GM, Sessle BJ (1993). The effect of bilateral cold block of the primate face primary somatosensory cortex on the performance of trained tongue-protrusion task and biting tasks. J Neurophysiol.

[46] Martin RE, Sessle BJ (1993). The role of the cerebral cortex in swallowing. Dysphagia.

[47] Murray GM, Lin LD, Moustafa EM, Sessle BJ (1991). Effects of reversible inactivation by cooling of the primate face motor cortex on the performance of a trained tongue-protrusion task and a trained biting task. J Neurophysiol.

[48] Sessle BJ (2006). Mechanisms of oral somatosensory and motor functions and their clinical correlates. J Oral Rehabil.

[49] Yao D, Yamamura K, Narita N, Murray GM, Sessle BJ (2002). Effects of reversible cold block of face primary somatosensory cortex on orofacial movements and related face primary motor cortex neuronal activity. Somatosens Mot Res.

[50] Schimmel M, Leemann B, Herrmann FR, Kiliaridis S, Schnider A, Muller F (2011). Masticatory function and bite force in stroke patients. J Dent Res.

[51] Woo J, Xing F, Prince JL, Stone M, Gomez AD, Reese TG, Wedeen VJ, El Fakhri G (2021). A deep joint sparse non-negative matrix factorization framework for identifying the common and subject-specific functional units of tongue motion during speech. Med Image Anal.

[52] Gomez AD, Stone ML, Woo J, Xing F, Prince JL (2020). Analysis of fiber strain in the human tongue during speech. Comput Methods Biomech Biomed Eng.

[53] Stone M, Epstein MA, Iskarous K (2004). Functional segments in tongue movement. Clin Linguist Phon.

[54] Hannam AG, Stavness I, Lloyd JE, Fels S (2008). A dynamic model of jaw and hyoid biomechanics during chewing. J Biomech.

[55] Stavness I, Lloyd JE, Fels S (2012). Automatic prediction of tongue muscle activations using a finite element model. J Biomech.

[56] Stavness I, Lloyd JE, Payan Y, Fels S (2011). Coupled hard-soft tissue simulation with contact and constraints applied to jaw–tongue–hyoid dynamics. Int J Numer Methods Biol.

[57] Jang H (2022). A tutorial on articulatory muscles and ArtiSynth: tongue and suprahyoid muscles, and 3D tongue model. Lang Linguist Compas.

[58] Yu J, Jiang C, Wang Z (2017). Creating and simulating a realistic physiological tongue model for speech production. Multimedia Tools Appl.

[59] Kappert KDR, Voskuilen L, Smeele LE, Balm AJM, Jasperse B, Nederveen AJ, van der Heijden F (2021). Personalized biomechanical tongue models based on diffusion-weighted MRI and validated using optical tracking of range of motion. Biomech Model Mechanobiol.

[60] Nakamura Y, Iriarte-Diaz J, Arce-McShane F, Orsbon CP, Brown KA, Eastment M, Avivi-Arber L, Sessle BJ, Inoue M, Hatsopoulos NG, Ross CF, Takahashi K (2017). Sagittal plane kinematics of the jaw and hyolingual apparatus during swallowing in *Macaca mulatta*. Dysphagia.

[61] Liu S, Iriarte-Diaz J, Hatsopoulos NG, Ross CF, Takahashi K, Chen Z (2019). Dynamics of motor cortical activity during naturalistic feeding behavior. J Neural Eng.

[62] Bollu T, Ito BS, Whitehead SC, Kardon B, Redd J, Liu MH, Goldberg JH (2021). Cortex-dependent corrections as the tongue reaches for and misses targets. Nature.

[63] Hiiemae KM, Palmer JB, Medicis SW, Hegener J, Jackson BS, Lieberman DE (2002). Hyoid and tongue surface movements in speaking and eating. Arch Oral Biol.

[64] Mayerl CJ, Steer KE, Chava AM, Bond LE, Edmonds CE, Gould FDH, Stricklen BM, Hieronymous TL, German RZ (2021). The contractile patterns, anatomy and physiology of the hyoid musculature change longitudinally through infancy. Proc Biol Sci.

[65] Bond LE, Mayerl CJ, Stricklen BM, German RZ, Gould FDH (2020). Changes in the coordination between respiration and swallowing from suckling through weaning. Biol Lett.

[66] Catchpole E, Bond L, German R, Mayerl C, Stricklen B, Gould FDH (2020). Reduced coordination of hyolaryngeal elevation and bolus movement in a pig model of preterm infant swallowing. Dysphagia.

[67] Edmonds CE, Catchpole EA, Gould FDH, Bond LE, Stricklen BM, German RZ, Mayerl CJ (2020). Preterm birth impacts the timing and excursion of oropharyngeal structures during infant feeding. Integr Org Biol.

[68] Mayerl CJ, Catchpole EA, Edmonds CE, Gould FDH, McGrattan KE, Bond LE, Stricklen BM, German RZ (2020). The effect of preterm birth, recurrent laryngeal nerve lesion, and postnatal maturation on hyoid and thyroid movements, and their coordination in infant feeding. J Biomech.

[69] Mayerl CJ, Edmonds CE, Catchpole EA, Myrla AM, Gould FDH, Bond LE, Stricklen BM, German RZ (2020). Sucking versus swallowing coordination, integration, and performance in preterm and term infants. J Appl Physiol.

[70] Mayerl CJ, Edmonds CE, Gould FDH, German RZ (2021). Increased viscosity of milk during infant feeding improves swallow safety through modifying sucking in an animal model. J Texture Stud.

[71] Mayerl CJ, Gould FDH, Bond LE, Stricklen BM, Buddington RK, German RZ (2019). Preterm birth disrupts the development of feeding and breathing coordination. J Appl Physiol.

[72] Mayerl CJ, Myrla AM, Bond LE, Stricklen BM, German RZ, Gould FDH (2020). Premature birth impacts bolus size and shape through nursing in infant pigs. Pediatr Res.

[73] Mayerl CJ, Myrla AM, Gould FDH, Bond LE, Stricklen BM, German RZ (2021). Swallow safety is determined by bolus volume during infant feeding in an animal model. Dysphagia.

[74] Mayerl CJ, Steer KE, Chava AM, Bond LE, Edmonds CE, Gould FDH, Hieronymous TL, Vinyard CJ, German RZ (2021). Anatomical and physiological variation of the hyoid musculature during swallowing in infant pigs. J Exp Biol.

[75] Almotairy N, Kumar A, Trulsson M, Grigoriadis A (2018). Development of the jaw sensorimotor control and chewing—a systematic review. Physiol Behav.

[76] Hatsopoulos NG, Donoghue JP (2009). The science of brain-machine iInterface technology. Annu Rev Neurosci.

[77] Luschei ES, Goldberg LJ, Brooks V (1981). Neural mechanisms of mandibular control: masication and voluntary biting. The nervous system motor control, part 2.

[78] Luschei ES, Goodwin GM (1974). Patterns of mandibular movement and jaw muscle activity during mastication in the monkey. J Neurophysiol.

[79] Luschei ES, Goodwin GM (1975). Role of monkey precentral cortex in control of voluntary jaw movements. J Neurophysiol.

[80] German RZ, Hertweck DW, Sirianni JE, Swindler DR (1994). Heterochrony and sexual dimorphism in the pigtailed macaque (*Macaca nemestrina*). Am J Phys Anthropol.

[81] German RZ, Saxe SA, Crompton AW, Hiiemae KM (1989). Food transport through the anterior oral cavity in macaques. Am J Phys Anthropol.

[82] Franks HA, Crompton AW, German RZ (1984). Mechanism of intraoral transport in macaques. Am J Phys Anthropol.

[83] Iriarte-Diaz J, Terhune CE, Taylor AB, Ross CF (2017). Functional correlates of the position of the axis of rotation of the mandible during chewing in non-human primates. Zoology (Jena).

[84] Hiiemae KM, Hayenga SM, Reese A (1995). Patterns of tongue and jaw movement in a cinefluorographic study of feeding in the macaque. Arch Oral Biol.

[85] Dechow PC, Carlson D (1990). Occlusal force and cramiofacial biomechanics during growth in rhesus monkeys. Am J Phys Anthropol.

[86] Dechow PC, Hylander WL (2000). Elastic properties amd masticatory bone stress in the macaque mandible. Am J Phys Anthropol.

[87] Panagiotopoulou O, Iriarte-Diaz J, Wilshin S, Dechow PC, Taylor AB, Mehari Abraha H, Aljunid SF, Ross CF (2017). In vivo bone strain and finite element modeling of a rhesus macaque mandible during mastication. Zoology (Jena).

[88] Dechow PC, Hylander WL (2000). Elastic properties and masticatory bone stress in the Macaque mandible. Am J Phys Anthropol.

[89] Hatanaka N, Tokuno H, Nambu A, Inoue T, Takada M (2005). Input-output organization of jaw-movement related areas in monkey frontal cortex. J Comp Neurol.

[90] Byrd KE, Milberg DJ, Luschei ES (1978). Human and macaque mastication: a quantitative study. J Dent Res.

[91] Morecraft RJ, Louie JL, Herrick JL, Stilwell-Morecraft KS (2001). Cortical innervation of the facial nucleus in the non-human primate—a new interpretation of the effects of stroke and related subtotal brain trauma on the muscles of facial expression. Brain.

[92] Morecraft RJ, Stilwell-Morecraft KS, Rossing WR (2004). The motor cortex and facial expression: new insights from neuroscience. Neurologist.

[93] Morecraft RJ, Stilwell-Morecraft KS, Solon-Cline KM, Ge JZ, Darling WG (2014). Cortical innervation of the hypoglossal nucleus in the non-human primate (*Macaca mulatta*). J Comp Neurol.

[94] Morecraft RJ, Binneboese A, Stilwell-Morecraft KS, Ge J (2017). Localization of orofacial representation in the corona radiata, internal capsule and cerebral peduncle in Macaca mulatta. J Comp Neurol.

[95] Hylander WL, Johnson KR, Crompton AW (1987). Loading patterns and jaw movements during mastication in *Macaca fascicularis*: a bone-strain, electromyographic, and cineradiographic analysis. Am J Phys Anthropol.

[96] Shaker R, Cook IJ, Dodds WJ, Hogan WJ, Arndorfer RC (1988). Pressure-flow dynamics of the oral phase of swallowing. Gastroenterology.

[97] Hori K, Ono T, Nokubi T (2006). Coordination of tongue pressure and jaw movement in mastication. J Dent Res.

[98] Anapol F (1988). Morphological and videofluorographic study of the hyoid apparatus and its function in the rabbit (*Oryctolagus cuniculus*). J Morphol.

[99] Thexton AJ, McGarrick JD (1988). Tongue movement of the cat during lapping. Arch Oral Biol.

[100] Thexton AJ, McGarrick JD (1989). Tongue movement in the cat during the intake of solid food. Arch Oral Biol.

[101] Thexton AJ, McGarrick JD (1994). The electromyographic activities of jaw and hyoid musculature in different ingestive behaviours in the cat. Arch Oral Biol.

[102] Laitman JT, Reidenberg JS (1993). Specializations of the human upper respiratory and upper digestive systems as seen through comparative and developmental anatomy. Dysphagia.

[103] Hogue AS, Ravosa MJ (2000). Mandibular symphyseal fusion in mammals: a test of competing hypotheses. Am Zool.

[104] Vinyard CJ, Ravosa MJ (1998). Ontogeny, function, and scaling of the mandibular symphysis in papionin primates. J Morphol.

[105] Scott JE, Hogue AS, Ravosa MJ (2012). The adaptive significance of mandibular symphyseal fusion in mammals. J Evol Biol.

[106] Thexton AJ (1984). Jaw, tongue and hyoid movement—a question of synchrony? Discussion paper. J R Soc Med.

[107] Williams SH, Bels V, Whishaw IQ (2019). Feeding in mammals: comparative, experimental, and evolutionary insights on form and function. Fascinat life science.

[108] Sanders I, Mu L (2013). A three-dimensional atlas of human tongue muscles. Anat Rec (Hoboken).

[109] Takemoto H (2001). Morphological analyses of the human tongue musculature for three-dimensional modeling. J Speech Lang Hear Res.

[110] Doran GA, Baggett H (1971). A structural and functional classification of mammalian tongues. J Mammal.

[111] Stone M, Woo J, Lee J, Poole T, Seagraves A, Chung M, Kim E, Murano EZ, Prince JL, Blemker SS (2018). Structure and variability in human tongue muscle anatomy. Comput Methods Biomech Biomed Eng Imaging Vis.

[112] Iwasaki SI, Yoshimura K, Shindo J, Kageyama I (2019). Comparative morphology of the primate tongue. Ann Anat.

[113] DePaul R, Abbs JH (1996). Quantitative morphology and histochemistry of intrinsic lingual muscle fibers in *Macaca fascicularis*. Acta Anat (Basel).

[114] Bosma JF (1956). Myology of the pharynx of cat, dog, and monkey with interpretation of the mechanism of swallowing. Ann Otol Rhinol Laryngol.

[115] Sanders I, Mu L, Amirali A, Su H, Sobotka S (2013). The human tongue slows down to speak: muscle fibers of the human tongue. Anat Rec (Hoboken).

[116] Berthommier F, Boë L-J, Meguerditchian A, Sawallis TR, Captier G, Louis-Jean B, Joël F, Pascal P, Jean-Luc S (2017). Comparative anatomy of the baboon and human vocal tracts: renewal of methods, data, and hypotheses. Origins of human language: continuities and discontinuities with nonhuman primates.

[117] Sokoloff AJ, Yang B, Li H, Burkholder TJ (2007). Immunohistochemical characterization of slow and fast myosin heavy chain composition of muscle fibres in the styloglossus muscle of the human and macaque (*Macaca rhesus*). Arch Oral Biol.

[118] Schwenk K, Brainerd EL, Crompton AW (1989). Biomechanics of the mammal tongue—role of the lingual tunic during hydrostatic deformation. Am Zool.

[119] Iwasaki SI (2002). Evolution of the structure and function of the vertebrate tongue. J Anat.

[120] Smith KK (1992). The evolution of the mammalian pharynx. Zool J Linn Soc-Lond.

[121] Li P, Ross CF, Luo ZX (2022). Morphological disparity and evolutionary transformations in the primate hyoid apparatus. J Hum Evol.

[122] Hilloowala RA (1975). Comparative anatomical study of the hyoid apparatus in selected primates. Am J Anat.

[123] Zhou CF, Bhullar BS, Neander AI, Martin T, Luo ZX (2019). New Jurassic mammaliaform sheds light on early evolution of mammal-like hyoid bones. Science.

[124] German RZ, Crompton AW, Thexton AJ (2004). The role of animal models in understanding feeding behavior in infants. Int J Orofacial Myol.

[125] Dimitrov R, Michaylov R, Ribarski S, Doichev V, Yordanova V (2014). Comparative morphology of the hyoid apparatus in wild boar (*Sus scrofa*) and domestic pig (*Sus scrofa domestica*). Philipp J Vet Med.

[126] Lesch R, Schwaha T, Orozco A, Shilling M, Brunelli S, Hofer M, Bowling DL, Zimmerberg B, Fitch WT (2021). Selection on vocal output affects laryngeal morphology in rats. J Anat.

[127] German RZ, Crompton AW, Thexton AJ (2009). Integration of the reflex pharyngeal swallow into rhythmic oral activity in a neurologically intact pig model. J Neurophysiol.

[128] Badhey A, Jategaonkar A, Anglin Kovacs AJ, Kadakia S, De Deyn PP, Ducic Y, Schantz S, Shin E (2017). Eagle syndrome: a comprehensive review. Clin Neurol Neurosurg.

[129] Graf W, Waele CD, Vidal PP (1995). Functional anatomy of the head-neck movement system of quadrupedal and bipedal mammals. J Anat.

[130] Vidal PP, Graf W, Berthoz A (1986). The orientation of the cervical vertebral column in unrestrained awake animals. Exp Brain Res.

[131] Steele J, Clegg M, Martelli S (2013). Comparative morphology of the hominin and African ape hyoid bone, a possible marker of the evolution of speech. Hum Biol.

[132] German RZ, Campbell-Malone R, Crompton AW, Ding P, Holman S, Konow N, Thexton AJ (2011). The concept of hyoid posture. Dysphagia.

[133] Plotsky K, Rendall D, Chase K, Riede T (2016). Cranio-facial remodeling in domestic dogs is associated with changes in larynx position. J Anat.

[134] Hellsing E (1989). Changes in the pharyngeal airway in relation to extension of the head. Eur J Orthod.

[135] McCluskie LK, Franklin SH, Lane JG, Tremaine WH, Allen KJ (2008). Effect of head position on radiographic assessment of laryngeal tie-forward procedure in horses. Vet Surg.

[136] Li P, Ross CF, Luo ZX, Gidmark NJ. Head posture and gape impact hyoid posture in mammals. In: Annual meeting of Society of Experimental Biology. Montpellier; 2022.

[137] Park JS, Hwang NK (2021). Chin tuck against resistance exercise for dysphagia rehabilitation: a systematic review. J Oral Rehabil.

[138] Balou M, McCullough GH, Aduli F, Brown D, Stack BC, Snoddy P, Guidry T (2014). Manometric measures of head rotation and chin tuck in healthy participants. Dysphagia.

[139] Welch MV, Logemann JA, Rademaker AW, Kahrilas PJ (1993). Changes in pharyngeal dimensions effected by chin tuck. Arch Phys Med Rehabil.

[140] Leigh JH, Oh BM, Seo HG, Lee GJ, Min Y, Kim K, Lee JC, Han TR (2015). Influence of the chin-down and chin-tuck maneuver on the swallowing kinematics of healthy adults. Dysphagia.

[141] Saconato M, Chiari BM, Lederman HM, Goncalves MI (2016). Effectiveness of chin-tuck maneuver to facilitate swallowing in neurologic dysphagia. Int Arch Otorhinolaryngol.

[142] Vanlunteren E, Haxhiu MA, Cherniack NS (1987). Relation between upper airway volume and hyoid muscle length. J Appl Physiol.

[143] Nishimura T, Matsuzawa T, Tomonaga M, Tanaka M (2006). Descent of the larynx in chimpanzees: mosaic and multile-step evolution of the foundations of human speech. Cognivtive development in chimpanzees.

[144] Lieberman DE, McCarthy RC, Hiiemae KM, Palmer JB (2001). Ontogeny of postnatal hyoid and larynx descent in humans. Arch Oral Biol.

[145] Sharma S, Patel R, Hashmi MF (2022). 3-3-2 rule.

[146] Fitch WT, Reby D (2001). The descended larynx is not uniquely human. Proc Biol Sci.

[147] Weissengruber GE, Forstenpointner G, Peters G, Kubber-Heiss A, Fitch WT (2002). Hyoid apparatus and pharynx in the lion (*Panthera leo*), jaguar (*Panthera onca*), tiger (*Panthera tigris*), cheetah (*Acinonyx jubatus*) and domestic cat (*Felis silvestris f. catus*). J Anat.

[148] Frey RA-OX, Reby D, Fritsch G, Charlton BD (2018). The remarkable vocal anatomy of the koala (*Phascolarctos cinereus*): insights into low-frequency sound production in a marsupial species. J Anat.

[149] Zeller U (1984). Zur Kenntnis des Stimmapparates der Epauletten Flughunde (Epomophorini, Pterpodidae, Megachiroptera). Z Säugetierkunde.

[150] Fitch WT, de Boer B, Mathur N, Ghazanfar AA (2016). Monkey vocal tracts are speech-ready. Sci Adv.

[151] Iwasaki SI, Yoshimura K, Asami T, Erdogan S (2022). Comparative morphology and physiology of the vocal production apparatus and the brain in the extant primates. Ann Anat.

[152] Dobson SD, Sherwood CC (2011). Mosaic evolution of brainstem motor nuclei in catarrhine primates. Anat Res Int.

[153] Sherwood CC, Hof PR, Holloway RL, Semendeferi K, Gannon PJ, Frahm HD, Zilles K (2005). Evolution of the brainstem orofacial motor system in primates: a comparative study of trigeminal, facial, and hypoglossal nuclei. J Hum Evol.

[154] Dunn JC, Smaers JB (2018). Neural correlates of vocal repertoire in primates. Front Neurosci.

[155] Vose A, Humbert I (2019). "Hidden in plain sight": a descriptive review of laryngeal vestibule closure. Dysphagia.

[156] Crompton AW, German RZ, Thexton A (1997). Mechanisms of swallowing and airway protection in infant mammals. J Zool (Lond).

[157] Crompton AW, German RZ, Thexton AJ (2008). Development of the movement of the epiglottis in infant and juvenile pigs. Zoology (Jena).

[158] Scapino RP (1976). Function of digastric muscle in carnivores. J Morphol.

[159] Curtis AA, Santana SE (2018). Jaw-dropping: functional variation in the digastric muscle in bats. Anat Rec (Hoboken).

[160] Standring S (2016). Gray's anatomy: the anatomical basis of clinical practice.

[161] Abd-el-Malek S (1955). The part played by the tongue in mastication and deglutition. J Anat.

[162] Tomura Y, Ide Y, Kamijo Y, Kawamura Y, Dubner R (1981). Studies on the morphological changes of the tongue movements during mastication by X-ray TV cinematography. Oral-facial sensory and motor functions.

[163] Mioche L, Hiiemae KM, Palmer JB (2002). A postero-anterior videofluorographic study of the intra-oral management of food in man. Arch Oral Biol.

[164] Schwartz DJ, Huelke DF (1963). Morphology of the head and neck of the macaque monkey: the muscles of mastication and the mandibular division of the trigeminal nerve. J Dent Res.

[165] Weijs WA (1973). Morphology of the muscles of mastication in the albina rat *Rattus norvegicus* (Berkenhout, 1769). Acta Morphol Neerland-Scand.

[166] Hiiemae KM, Houston WJB (1971). The structure and function of the jaw muscles in the rat (*Rattus norvegicus* L.). I. Their anatomy and internal architecture. Zool J Linn Soc-Lond.

[167] Turnbull WD (1970). Mammalian masticatory apparatus. Fieldiana Geol.

[168] Kletzien H, Cullins MJ, Connor NP (2019). Age-related alterations in swallowing biomechanics. Exp Gerontol.

[169] Cortopassi D, Muhl ZF (1990). Videofluorographic analysis of tongue movement in the rabbit (*Oryctolagus cuniculus*). J Morphol.

[170] Takata H (1989). A comparative anatomical study on suprahyoid muscles. Journal of Kyushu Dental Society.

[171] Gaspard M (1971). Anatomie comparative et fonctionelle de la musculature masticatrice chew les carnivores.

[172] Evans HE, Miller ME (2013). Miller's anatomy of the dog.

[173] Watrous BJ, Suter PF (1979). Normal swallowing in the dog—cineradiographic study. Vet Radiol.

[174] Kobara-Mates M, Logemann JA, Larson C, Kahrilas PJ (1995). Physiology of oropharyngeal swallow in the cat: a videofluoroscopic and electromyographic study. Am J Physiol.

[175] Magalhaes HIR, Barcelos JB, Romao FB, Borges TRJ, de Carvalho-Barros RA, Miglino MA, Silva FOCE, Ribeiro LD (2021). Comparative study of the digastric and the stylohyoid muscles between wild boars (*Sus scrofa scrofa*) and domestic swine (*Sus scrofa domesticus*): revisiting the gross anatomy. Anat Cell Biol.

[176] Herring SW, Scapino RP (1974). Physiology of feeding in miniature pigs. J Morphol.

[177] Thexton AJ, Crompton AW, German RZ (1998). Transition from suckling to drinking at weaning: a kinematic and electromyographic study in miniature pigs. J Exp Zool.

[178] Hiiemae KM, Jenkins FA (1969). The anatomy and internal architecture of the muscles of mastication in *Didelphis marsupialis*. Postilla.

[179] Hiiemae KM, Thexton AJ, Crompton AW, Carlson DS, McNamara JA (1978). Intra-oral food transport: the fundamental mechanism of feeding. Muscle adaptation in the Craniofacial Region.

[180] Jacobs R, Serhal CB, Van Steenberghe D (1998). Oral stereognosis: a review of the literature. Clin Oral Investig.

[181] Sivapathasundharam B, Biswas PG (2020). Oral stereognosis—a literature review. Eur J Mol Clin Med.

[182] Lucas PW, Prinz JF, Agrawal KR (2002). Food physics and oral physiology. Food Qual Prefer.

[183] Humbert IA, Lokhande A, Christopherson H, German R, Stone A (2012). Adaptation of swallowing hyo-laryngeal kinematics is distinct in oral vs pharyngeal sensory processing. J Appl Physiol.

[184] Hiiemae KM, Crompton AW, Hildebrand M, Bramble DM, Liem KF, Wake DB (1985). Mastication, food transport, and swallowing. Functional vertebrate morphology.

[185] Ross CF, Iriarte-Diaz J (2014). What does feeding system morphology tell us about feeding?. Evol Anthropol.

[186] Ross CF, Washington RL, Eckhardt A, Reed DA, Vogel ER, Dominy NJ, Machanda ZP (2009). Ecological consequences of scaling of chew cycle duration and daily feeding time in primates. J Hum Evol.

[187] Iriarte-Diaz J, Reed DA, Ross CF (2011). Sources of variance in temporal and spatial aspects of jaw kinematics in two species of primates feeding on foods of different properties. Integr Comp Biol.

[188] Hiiemae KM, Heath MR, Heath G, Kazazoglu E, Murray J, Sapper D, Hamblett K (1996). Natural bites, food consistency and feeding behaviour in man. Arch Oral Biol.

[189] Hiiemae KM, Palmer JB (1999). Food transport and bolus formation during complete feeding sequences on foods of different initial consistency. Dysphagia.

[190] Franks HA, German RZ, Crompton AW, Hiiemae KM (1985). Mechanism of intra-oral transport in a herbivore, the hyrax (*Procavia syriacus*). Arch Oral Biol.

[191] De Gueldre G, De Vree F (1984). Movements of the mandibles and tongue during mastication and swallowing in *Pteropus giganteus* (Megachiroptera): a cineradiographical study. J Morphol.

[192] Montuelle SJ, Olson RA, Curtis H, Sidote JV, Williams SH (2019). The effect of unilateral lingual nerve injury on the kinematics of mastication in pigs. Arch Oral Biol.

[193] Laurence-Chasen JD, Manafzadeh AR, Hatsopoulos NG, Ross CF, Arce-McShane FI (2020). Integrating XMALab and DeepLabCut for high-throughput XROMM. J Exp Biol.

[194] Bramble DM, Wake DB, Hildebrand M, Bramble DM, Liem KF, Wake DB (1985). Feeding mechanisms in lower tetrapods. Functional vertebrate morphology.

[195] Ross CF, Iriarte-Diaz J, Bels V (2019). Evolution, constraint and optimality in primate feeding systems. Feeding in vertebrates.

[196] Lund JP (1991). Mastication and its control by the brain stem. Crit Rev Oral Biol Med.

[197] Zelditch ML, Swiderski DL, Sheets HD (2012). Geometric morphometrics for biologists: a primer.

[198] Tran TTA, Harris BM, Pearson WG (2018). Improvements resulting from respiratory-swallow phase training visualized in patient-specific computational analysis of swallowing mechanics. Comput Methods Biomech Biomed Eng Imaging Vis.

[199] Livingstone R (1956). Some observations on the natural history of the tongue. Ann R Coll Surg Engl.

[200] Hiiemae KM, Schwenk K (2000). Feeding in mammals. feeding: form, function and evolution in tetrapod vertebrates.

[201] Liu ZJ, Kayalioglu M, Shcherbatyy V, Seifi A (2007). Tongue deformation, jaw movement and muscle activity during mastication in pigs. Arch Oral Biol.

[202] Kayalioglu M, Shcherbatyy V, Seifi A, Liu ZJ (2007). Roles of intrinsic and extrinsic tongue muscles in feeding: electromyographic study in pigs. Arch Oral Biol.

[203] Hiiemae KM, Chivers DJ, Wood BA, Bilsborough A (1984). Functional aspects of primate jaw morphology. Food acquisition and processing in primates.

[204] McClung JR, Goldberg SJ (2000). Functional anatomy of the hypoglossal innervated muscles of the rat tongue: a model for elongation and protrusion of the mammalian tongue. Anat Rec.

[205] Palmer JB, Hiiemae KM, Liu J (1997). Tongue-jaw linkages in human feeding: a preliminary videofluorographic study. Arch Oral Biol.

[206] Liu ZJ, Shcherbatyy V, Kayalioglu M, Seifi A (2009). Internal kinematics of the tongue in relation to muscle activity and jaw movement in the pig. J Oral Rehabil.

[207] Taniguchi H, Matsuo K, Okazaki H, Yoda M, Inokuchi H, Gonzalez-Fernandez M, Inoue M, Palmer JB (2013). Fluoroscopic evaluation of tongue and jaw movements during mastication in healthy humans. Dysphagia.

[208] Palmer JB, Rudin NJ, Lara GL, Crompton AW (1992). Coordination of mastication and swallowing. Dysphagia.

[209] Matsuo A, Palmer J, Shaker R, Belafsky P, Postma G, Easterling C (2013). Oral phase preparation and propulsion: anatomy, physiology, rheology, mastication, and transport. Principles of deglutition: a multidisciplinary text for swallowing and its disorders.

[210] Winters TM, Sepulveda GS, Cottler PS, Kaufman KR, Lieber RL, Ward SR (2009). Correlation between isometric force and intramuscular pressure in rabbit tibialis anterior muscle with an intact anterior compartment. Muscle Nerve.

[211] Siebert T, Till O, Stutzig N, Gunther M, Blickhan R (2014). Muscle force depends on the amount of transversal muscle loading. J Biomech.

[212] Azizi E, Deslauriers AR, Holt NC, Eaton CE (2017). Resistance to radial expansion limits muscle strain and work. Biomech Model Mechanobiol.

[213] Ruttiman RJ, Sleboda DA, Roberts TJ (1985). Release of fascial compartment boundaries reduces muscle force output. J Appl Physiol.

[214] Sleboda DA, Roberts TJ (2020). Internal fluid pressure influences muscle contractile force. Proc Natl Acad Sci USA.

[215] Kier WM, Smith KK (1985). Tongues, tentacles and trunks—the biomechanics of movement in muscular-hydrostats. Zool J Linn Soc-Lond.

[216] Gilbert RJ, Napadow VJ, Gaige TA, Wedeen VJ (2007). Anatomical basis of lingual hydrostatic deformation. J Exp Biol.

[217] Smith KK, Kier WM (1989). Trunks, tongues, and tentacles—moving with skeletons of muscle. Am Sci.

[218] Napadow VJ, Chen Q, Wedeen VJ, Gilbert RJ (1999). Biomechanical basis for lingual muscular deformation during swallowing. Am J Physiol.

[219] Napadow VJ, Chen Q, Wedeen VJ, Gilbert RJ (1999). Intramural mechanics of the human tongue in association with physiological deformations. J Biomech.

[220] Doty RW, Bosma JF (1956). An electromyographic analysis of reflex deglutition. J Neurophysiol.

[221] Gassert RB, Pearson WG (2016). Evaluating muscles underlying tongue base retraction in deglutition using muscular functional magnetic resonance imaging (mfMRI). Magn Reson Imaging.

[222] Konow N, Thexton A, Crompton AW, German RZ (2010). Regional differences in length change and electromyographic heterogeneity in sternohyoid muscle during infant mammalian swallowing. J Appl Physiol.

[223] Kendall KA, McKenzie SW, Leonard RJ, Jones CU (2000). Timing of swallowing events after single-modality treatment of head and neck carcinomas with radiotherapy. Ann Otol Rhinol Laryngol.

[224] Kendall KA, McKenzie SW, Leonard RJ, Jones C (1998). Structural mobility in deglutition after single modality treatment of head and neck carcinomas with radiotherapy. Head Neck.

[225] May NH, Pisegna JM, Marchina S, Langmore SE, Kumar S, Pearson WG (2017). Pharyngeal swallowing mechanics secondary to hemispheric stroke. J Stroke Cerebrovasc Dis.

[226] Knigge MA, Thibeault S (2016). Relationship between tongue base region pressures and vallecular clearance. Dysphagia.

[227] Takagi D, Ohno T, Moriwaki M, Katagiri N, Umeda Y, Tohara H, Nomoto A, Fujishima I (2021). Effect of dentures on pharyngeal swallowing function in patients with dysphagia. Geriatr Gerontol Int.

[228] Ekberg O (1986). The normal movements of the hyoid bone during swallow. Investig Radiol.

[229] Ishida R, Palmer JB, Hiiemae KM (2002). Hyoid motion during swallowing: factors affecting forward and upward displacement. Dysphagia.

[230] Langmore SE. Endoscopic evaluation of oral and pharyngeal phases of swallowing. GI Motility Online. 2006. 10.1038/gimo28

[231] McNamara JA, Moyers RE (1973). Electromyography of the oral phase of deglutition in the Rhesus monkey (*Macaca mulatta*). Arch Oral Biol.

[232] Inokuchi H, Gonzalez-Fernandez M, Matsuo K, Brodsky MB, Yoda M, Taniguchi H, Okazaki H, Hiraoka T, Palmer JB (2014). Electromyography of swallowing with fine wire intramuscular electrodes in healthy human: activation sequence of selected hyoid muscles. Dysphagia.

[233] Kawagishi S. The stereognostic ability of the tongue and its role in eating and swallowing. Tongue Anat Kinemat Dis. 2012; 37–52.

[234] Moayedi Y, Michlig S, Park M, Koch A, Lumpkin EA (2021). Somatosensory innervation of healthy human oral tissues. J Comp Neurol.

[235] Maeyama T, Plattig KH (1989). Minimal 2-point discrimination in human tongue and palate. Am J Otolaryngol.

[236] Lozano CA, Kaczmarek KA, Santello M (2009). Electrotactile stimulation on the tongue: intensity perception, discrimination, and cross-modality estimation. Somatosens Mot Res.

[237] Wilson JA, Walton LM, Tyler M, Williams J (2012). Lingual electrotactile stimulation as an alternative sensory feedback pathway for brain-computer interface applications. J Neural Eng.

[238] Miles BL, Van Simaeys K, Whitecotton M, Simons CT (2018). Comparative tactile sensitivity of the fingertip and apical tongue using complex and pure tactile tasks. Physiol Behav.

[239] Kubota K, Negishi T, Masegi T (1975). Topological distribution of muscle spindles in the human tongue and its significance in proprioception. Bull Tokyo Med Dent Univ.

[240] Cooper S (1953). Muscle spindles in the intrinsic muscles of the human tongue. J Physiol Lond.

[241] Saigusa H, Yamashita K, Tanuma K, Saigusa M, Niimi S (2004). Morphological studies for retrusive movement of the human adult tongue. Clin Anat.

[242] Liss JM (1990). Muscle spindles in the human levator veli palatini and palatoglossus muscles. J Speech Hear Res.

[243] Kuehn DP, Templeton PJ, Maynard JA (1990). Muscle spindles in the velopharyngeal musculature of humans. J Speech Hear Res.

[244] Kubota K, Masegi T (1977). Muscle spindle supply to the human jaw muscle. J Dent Res.

[245] Saverino D, De Santanna A, Simone R, Cervioni S, Cattrysse E, Testa M (2014). Observational study on the occurrence of muscle spindles in human digastric and mylohyoideus muscles. Biomed Res Int.

[246] de Carlos F, Cobo J, Macias E, Feito J, Cobo T, Calavia MG, Garcia-Suarez O, Vega JA (2013). The sensory innervation of the human pharynx: searching for mechanoreceptors. Anat Rec (Hoboken).

[247] Fitzgerald MJT, Sachithanandan SR (1979). Structure and source of lingual proprioceptors in the monkey. J Anat.

[248] Bowman JP (1968). Muscle spindles in intrinsic and extrinsic muscles of rhesus monkeys (*Macaca mulatta*) tongue. Anat Rec.

[249] Sengupta BN, Sengupta S (1978). Muscle spindles in the inferior constrictor pharyngis muscle of the crab-eating monkey (*Macaca irus*). Acta Anat (Basel).

[250] Smith KK (1989). Histological demonstration of muscle-spindles in the tongue of the rat. Arch Oral Biol.

[251] Muhl ZF, Kotov O (1988). Muscle-spindles in the digastric muscle of the rabbit. J Dent Res.

[252] Ross CF, Baden AL, Georgi JA, Herrel A, Metzger KA, Reed DA, Schaerlaeken V, Wolff MS (2010). Chewing variation in lepidosaurs and primates. J Exp Biol.

[253] Ross CF, Eckhardt A, Herrel A, Hylander WL, Metzger KA, Schaerlaeken V, Washington RL, Williams SH (2007). Modulation of intra-oral processing in mammals and lepidosaurs. Integr Comp Biol.

[254] Thexton AJ, Hiiemae KM, Crompton AW (1980). Food consistency and bite size as regulators of jaw movement during feeding in the cat. J Neurophysiol.

[255] Thexton AJ, Crompton AW (1989). Effect of sensory input from the tongue on jaw movement in normal feeding in the opossum. J Exp Zool.

[256] Schwartz G, Enomoto S, Valiquette C, Lund JP (1989). Mastication in the rabbit: a description of movement and muscle activity. J Neurophysiol.

[257] Miller MG (1981). Trigeminal deafferentation and ingestive behavior in rats. J Comp Physiol Psychol.

[258] Zeigler HP, Jacquin MF, Miller MG (1984). Trigeminal sensorimotor mechanisms and ingestive behavior. Neurosci Biobehav Rev.

[259] Inoue T, Kato T, Masuda Y, Nakamura T, Kawamura Y, Morimoto T (1989). Modifications of masticatory behavior after trigeminal deafferentation in the rabbit. Exp Brain Res.

[260] Huang X, Zhang G, Herring SW (1993). Effects of oral sensory afferents on mastication in the miniature pig. J Dent Res.

[261] Montuelle SJ, Olson RA, Curtis H, Williams SH (2020). Unilateral lingual nerve transection alters jaw-tongue coordination during mastication in pigs. J Appl Physiol.

[262] Engelen L, van der Bilt A, Bosman F (2004). Relationship between oral sensitivity and masticatory performance. J Dent Res.

[263] Maruyama M, Morita K, Kimura H, Nishio F, Yoshida M, Tsuga K (2020). Association between masticatory ability and oral functions. J Clin Exp Dent.

[264] Koshino H, Hirai T, Ishijima T, Ikeda Y (1997). Tongue motor skills and masticatory performance in adult dentates, elderly dentates, and complete denture wearers. J Prosthet Dent.

[265] Miles BA-O, Wu Z, Kennedy KS, Zhao K, Simons CA-O (2022). Elucidation of a lingual detection mechanism for high-viscosity solutions in humans. Food Funct.

[266] Penfield W, Rasmussen T (1950). The cerebral cortex of man; a clinical study of localization of function.

[267] Miller AJ, Bowman JP (1977). Precentral cortical modulation of mastication and swallowing. J Dent Res.

[268] Hamdy S, Aziz Q, Rothwell JC, Singh KD, Barlow J, Hughes DG, Tallis RC, Thompson DG (1996). The cortical topography of human swallowing musculature in health and disease. Nat Med.

[269] Hamdy S, Mikulis DJ, Crawley A, Xue SW, Lau H, Henry S, Diamant NE (1999). Cortical activation during human volitional swallowing: an event-related fMRI study. Am J Physiol-Gastrointest Liver Physiol.

[270] Mosier KM, Liu WC, Maldjian JA, Shah R, Modi B (1999). Lateralization of cortical function in swallowing: a functional MR imaging study. Am J Neuroradiol.

[271] Martin RE, Goodyear BG, Gati JS, Menon RS (2001). Cerebral cortical representation of automatic and volitional swallowing in humans. J Neurophysiol.

[272] Martin RE, MacIntosh BJ, Smith RC, Barr AM, Stevens TK, Gati JS, Menon RS (2004). Cerebral areas processing swallowing and tongue movement are overlapping but distinct: a functional magnetic resonance imaging study. J Neurophysiol.

[273] Soros P, Inamoto Y, Martin RE (2009). Functional brain imaging of swallowing: an activation likelihood estimation meta-analysis. Hum Brain Mapp.

[274] Toogood JA, Barr AM, Stevens TK, Gati JS, Menon RS, Martin RE (2005). Discrete functional contributions of cerebral cortical foci in voluntary swallowing: a functional magnetic resonance imaging (fMRI) "Go, No-Go" study. Exp Brain Res.

[275] Humbert IA, Robbins J (2007). Normal swallowing and functional magnetic resonance imaging: a systematic review. Dysphagia.

[276] Dziewas R, Soros P, Ishii R, Chau W, Henningsen H, Ringelstein EB, Knecht S, Pantev C (2003). Neuroimaging evidence for cortical involvement in the preparation and in the act of swallowing. Neuroimage.

[277] Robbins J, Levin RL (1988). Swallowing after unilateral stroke of the cerebral cortex: preliminary experience. Dysphagia.

[278] Avivi-Arber L, Lee JC, Sessle BJ (2011). Chapter 9—face sensorimotor cortex neuroplasticity associated with intraoral alterations. Prog Brain Res.

[279] Avivi-Arber L, Martin R, Lee JC, Sessle BJ (2011). Face sensorimotor cortex and its neuroplasticity related to orofacial sensorimotor functions. Arch Oral Biol.

[280] Arce-McShane FI, Hatsopoulos NG, Lee JC, Ross CF, Sessle BJ (2014). Modulation dynamics in the orofacial sensorimotor cortex during motor skill acquisition. J Neurosci.

[281] Arce-McShane FI, Ross CF, Takahashi K, Sessle BJ, Hatsopoulos NG (2016). Primary motor and sensory cortical areas communicate via spatiotemporally coordinated networks at multiple frequencies. Proc Natl Acad Sci USA.

[282] Arce-McShane FI, Sessle BJ, Ross CF, Hatsopoulos NG (2018). Primary sensorimotor cortex exhibits complex dependencies of spike-field coherence on neuronal firing rates, field power, and behavior. J Neurophysiol.

[283] Toda T, Taoka M (2001). The complexity of receptive fields of periodontal mechanoreceptive neurons in the postcentral area 2 of conscious macaque monkey brains. Arch Oral Biol.

[284] Toda T, Taoka M (2002). Hierarchical somesthetic processing of tongue inputs in the postcentral somatosensory cortex of conscious macaque monkeys. Exp Brain Res.

[285] Toda T, Taoka M (2004). Converging patterns of inputs from oral structures in the postcentral somatosensory cortex of conscious macaque monkeys. Exp Brain Res.

[286] Toda T, Taoka M (2006). Postcentral neurons with covert receptive fields in conscious macaque monkeys: their selective responsiveness to simultaneous two-point stimuli applied to discrete oral portions. Exp Brain Res.

[287] Huang CS, Sirisko MA, Hiraba H, Murray GM, Sessle BJ (1988). Organization of the primate face motor cortex as revealed by intracortical microstimulation and electrophysiological identification of afferent inputs and corticobulbar projections. J Neurophysiol.

[288] Park MC, Belhaj-Saif A, Gordon M, Cheney PD (2001). Consistent features in the forelimb representation of primary motor cortex in rhesus macaques. J Neurosci.

[289] Nudo RJ, Jenkins WM, Merzenich MM, Prejean T, Gedela R (1992). Neurophysiological correlates of hand preference in primary motor cortex of squirrel monkey. J Neurosci.

[290] Martin RE, Kemppainen P, Masuda Y, Yao D, Murray GM, Sessle BJ (1999). Features of cortically evoked swallowing in the awake primate (*Macaca fascicularis*). J Neurophysiol.

[291] Murray GM, Sessle BJ (1992). Functional properties of single neurons in the face primary motor cortex of the primate. III. Relations with different directions of trained tongue protrusion. J Neurophysiol.

[292] Murray GM, Sessle BJ (1992). Functional properties of single neurons in the face primary motor cortex of the primate. I. Input and output features of tongue motor cortex. J Neurophysiol.

[293] Murray GM, Sessle BJ (1992). Functional properties of single neurons in the face primary motor cortex of the primate. II. Relations with trained orofacial motor behavior. J Neurophysiol.

[294] Lin LD, Murray GM, Sessle BJ (1994). Functional properties of single neurons in the primate face primary somatosensory cortex. I. Relations with trained orofacial motor behaviors. J Neurophysiol.

[295] Lin LD, Murray GM, Sessle BJ (1994). Functional properties of single neurons in the primate face primary somatosensory cortex. II. Relations with different directions of trained tongue protrusion. J Neurophysiol.

[296] Martin RE, Murray GM, Kemppainen P, Masuda Y, Sessle BJ (1997). Functional properties of neurons in the primate tongue primary motor cortex during swallowing. J Neurophysiol.

[297] Arce FI, Lee JC, Ross CF, Sessle BJ, Hatsopoulos NG (2013). Directional information from neuronal ensembles in the primate orofacial sensorimotor cortex. J Neurophysiol.

[298] Nogles TE, Galuska MA (2022). Middle cerebral artery stroke.

[299] Martino R, Foley N, Bhogal S, Diamant N, Speechley M, Teasell R (2005). Dysphagia after stroke—incidence, diagnosis, and pulmonary complications. Stroke.

[300] Dziewas R, Ritter M, Schilling M, Konrad C, Oelenberg S, Nabavi DG, Stogbauer F, Ringelstein EB, Ludemann P (2004). Pneumonia in acute stroke patients fed by nasogastric tube. J Neurol Neurosur Psychiatry.

[301] Dziewas R, Stellato R, van der Tweel I, Walther E, Werner CJ, Braun T, Citerio G, Jandl M, Friedrichs M, Nötzel K, Vosko MR, Mistry S, Hamdy S, McGowan S, Warnecke T, Zwittag P, Bath PM, Vosko M, Aroyo I, Bucka C, Kerz T, Köstenberger M, Marschner-Preuth N, Niesen W-D, Pfausler B (2018). Pharyngeal electrical stimulation for early decannulation in tracheotomised patients with neurogenic dysphagia after stroke (PHAST-TRAC): a prospective, single-blinded, randomised trial. Lancet Neurol.

[302] Cohen DL, Roffe C, Beavan J, Blackett B, Fairfield CA, Hamdy S, Havard D, McFarlane M, McLauglin C, Randall M, Robson K, Scutt P, Smith C, Smithard D, Sprigg N, Warusevitane A, Watkins C, Woodhouse L, Bath PM (2016). Post-stroke dysphagia: a review and design considerations for future trials. Int J Stroke.

[303] Nishioka S, Okamoto T, Takayama M, Urushihara M, Watanabe M, Kiriya Y, Shintani K, Nakagomi H, Kageyama N (2017). Malnutrition risk predicts recovery of full oral intake among older adult stroke patients undergoing enteral nutrition: secondary analysis of a multicentre survey (the APPLE study). Clin Nutr.

[304] Kim DY, Park HS, Park SW, Kim JH (2020). The impact of dysphagia on quality of life in stroke patients. Medicine.

[305] Bartolo A, Ham HS (2016). A cognitive overview of limb apraxia. Curr Neurol Neurosci Rep.

[306] Heilman KM, Rothi LJ, Valenstein E (1982). Two forms of ideomotor apraxia. Neurology.

[307] Robbins J, Levine RL, Maser A, Rosenbek JC, Kempster GB (1993). Swallowing after unilateral stroke of the cerebral cortex. Arch Phys Med Rehabil.

[308] Daniels SK, Foundas AL (1997). The role of the insular cortex in dysphagia. Dysphagia.

[309] Green HD, Walker AE (1938). The effects of ablation of the cortical motor face area in monkeys. J Neurophysiol.

[310] Larson CR, Byrd KE, Garthwaite CR, Luschei ES (1980). Alterations in the pattern of mastication after ablations of the lateral precentral cortex in rhesus macaques. Exp Neurol.

[311] Lin LD, Murray GM, Sessle BJ (1998). Effects on non-human primate mastication of reversible inactivation by cooling of the face primary somatosensory cortex. Arch Oral Biol.

[312] Yamamura K, Narita N, Yao D, Martin RE, Masuda Y, Sessle BJ (2002). Effects of reversible bilateral inactivation of face primary motor cortex on mastication and swallowing. Brain Res.

[313] Narita N, Yamamura K, Yao D, Martin RE, Masuda Y, Sessle BJ (2002). Effects on mastication of reversible bilateral inactivation of the lateral pericentral cortex in the monkey (*Macaca fascicularis*). Arch Oral Biol.

[314] Narita N, Yamamura K, Yao D, Martin RE, Sessle BJ (1999). Effects of functional disruption of lateral pericentral cerebral cortex on primate swallowing. Brain Res.

[315] Enomoto S, Schwartz G, Lund JP (1987). The effects of cortical ablation on mastication in the rabbit. Neurosci Lett.

[316] Gignac PM, Kley NJ, Clarke JA, Colbert MW, Morhardt AC, Cerio D, Cost IN, Cox PG, Daza JD, Early CM, Echols MS, Henkelman RM, Herdina AN, Holliday CM, Li Z, Mahlow K, Merchant S, Muller J, Orsbon CP, Paluh DJ, Thies ML, Tsai HP, Witmer LM (2016). Diffusible iodine-based contrast-enhanced computed tomography (diceCT): an emerging tool for rapid, high-resolution, 3-D imaging of metazoan soft tissues. J Anat.

[317] Beck PD, Pospichal MW, Kaas JH (1996). Topography, architecture, and connections of somatosensory cortex in opossums: evidence for five somatosensory areas. J Comp Neurol.

